# Coordinated Evolution of Influenza A Surface Proteins

**DOI:** 10.1371/journal.pgen.1005404

**Published:** 2015-08-06

**Authors:** Alexey D. Neverov, Sergey Kryazhimskiy, Joshua B. Plotkin, Georgii A. Bazykin

**Affiliations:** 1 Central Research Institute for Epidemiology, Moscow, Russia; 2 Department of Organismic and Evolutionary Biology, Harvard University, Cambridge, Massachusetts, United States of America; 3 FAS Center for Systems Biology, Harvard University, Cambridge, Massachusetts, United States of America; 4 Department of Biology, University of Pennsylvania, Philadelphia, Pennsylvania, United States of America; 5 Institute for Information Transmission Problems (Kharkevich Institute) of the Russian Academy of Sciences, Moscow, Russia; 6 Faculty of Bioengineering and Bioinformatics, Lomonosov Moscow State University, Moscow, Russia; 7 Belozersky Institute of Physico-Chemical Biology, Lomonosov Moscow State University, Moscow, Russia; 8 Pirogov Russian National Research Medical University, Moscow, Russia; Fred Hutchinson Cancer Research Center, UNITED STATES

## Abstract

The surface proteins hemagglutinin (HA) and neuraminidase (NA) of human influenza A virus evolve under selection pressures to escape adaptive immune responses and antiviral drug treatments. In addition to these external selection pressures, some mutations in HA are known to affect the adaptive landscape of NA, and vice versa, because these two proteins are physiologically interlinked. However, the extent to which evolution of one protein affects the evolution of the other one is unknown. Here we develop a novel phylogenetic method for detecting the signatures of such genetic interactions between mutations in different genes – that is, inter-gene epistasis. Using this method, we show that influenza surface proteins evolve in a coordinated way, with mutations in HA affecting subsequent spread of mutations in NA and vice versa, at many sites. Of particular interest is our finding that the oseltamivir-resistance mutations in NA in subtype H1N1 were likely facilitated by prior mutations in HA. Our results illustrate that the adaptive landscape of a viral protein is remarkably sensitive to its genomic context and, more generally, that the evolution of any single protein must be understood within the context of the entire evolving genome.

## Author Summary

The fitness of an organism depends on the coordinated function of many genes. Thus, how a mutation in one gene affects fitness often depends on what mutations are present in other genes. This dependence is called “genetic interaction” or “epistasis”. The prevalence and type of such interactions are not well understood. Epistasis can be inferred from time-series sequencing data when a mutation in one gene is observed to facilitate the spread of a mutation in another gene. However, the situation is much more complicated when new combinations of genes are formed by processes such as recombination or reassortment. In such cases, deducing the time and order of genetic changes is difficult. Here, we devise a method to infer pairs of mutations in different genes which closely follow one another in the presence of reassortment. We apply it to evolution of two surface proteins of influenza A virus, hemagglutinin and neuraminidase, which are important targets for the human immune system and drugs. We show that mutations in one of these proteins are often facilitated by prior mutations, or compensated by subsequent mutations, in the other protein. In particular, drug-resistance mutations in neuraminidase were likely made possible by prior mutation in hemagglutinin. Knowledge of such interactions is necessary to fully understand and predict evolution.

## Introduction

One of the central obstacles in controlling many pathogen-borne diseases is their exceptional ability to adapt through evolutionary changes [1]. Large population sizes and high mutation rates in many pathogens make them extremely effective at evolving to evade the immune system or resist drug treatments [2–6]. Our ability to prevent or even predict such escape mutations is hampered by limited knowledge of the effects of new mutations on pathogen fitness. This problem is made especially difficult because the effect of any particular mutation is often dependent on the genetic background in which it occurs, a phenomenon called epistasis [7–16].

Epistasis is particularly common among mutations that arise in response to strong selection pressures. For example, resistance mutations that arise under drug treatments often carry substantial fitness costs which are alleviated by secondary, compensatory, mutations [7,10,14–16]. Likewise, mutations that facilitate immune escape are in several cases known to be epistatic with other, compensatory or permissive, mutations [17,18]. The surface proteins hemagglutinin (HA) and neuraminidase (NA) of the human influenza A virus evolve under strong selection pressures imposed by the human immune system and, possibly, antiviral drugs [4,19]. It is therefore expected that epistasis may play an important role in the evolution of these proteins. Several previous studies have found that epistasis within each of these proteins is widespread, so that mutations in a given protein are often beneficial only in the presence of mutations at other sites in the same protein [19–21].

Aside from intra-gene epistasis, we also might expect inter-gene epistasis, especially in the case of the HA and NA proteins of influenza viruses, which serve complementary physiological functions. HA facilitates the attachment of the virus to the cell surface, whereas NA catalyzes the separation of the ready-made virus particles from the cell. Thus, mutations that increase receptor-binding avidity of HA should promote mutations in NA that increase its cleavage activity [22,23] and vice versa [24,25]. HA and NA jointly determine sensitivity to neuraminidase inhibitors, with mutations in HA compensating for the reduction in binding affinity of NA caused by the inhibitors [26,27]. Other, as yet unknown, molecular interaction mechanisms may also lead to inter-gene epistasis. Indirect evidence also suggests that interactions between HA and NA may be strong; for example, reassortments giving rise to new combinations of HA and NA lead to a temporary increase in the rate of accumulation of mutations in these genes, likely due to changes adjusting the genes to each other [28,29].

Here we present a method toWe set out to detect signatures of inter-gene epistasis, and apply it to understand the evolutionary history of influenza surface proteins. The method we develop is an extension of techniques previously developed for detecting intra-gene epistasis [21,30]. The idea behind it is simple: epistasis will tend to induce temporal clustering of mutations along the phylogeny of an adapting protein, with mutations at one site followed rapidly by mutations at another, interacting site. In the case of mutations within a single protein it is straightforward to develop this idea into a rigorous statistical test, by quantifying the time that separates subsequent mutations along the protein’s phylogeny. All the sites within a single influenza protein share a common phylogenetic history: recombination events within an influenza virus RNA segment are exceedingly rare [31], and so sites that reside on the same segment of the viral genome are completely linked. However, influenza viruses undergo frequent reassortment events, so that sites residing on different segments typically have different genealogies—a complication that obscures the temporal order of mutations occurring on different RNA segments. To resolve this complication, here we develop a method for inferring the relative temporal order of mutations at sites that have different evolutionary histories, and then use this information to detect temporal clustering of such mutations in influenza viruses. We find that origination of mutant alleles at many sites in NA facilitated the spread of subsequent mutations in HA and vice versa, implying that inter-gene epistasis has shaped the molecular evolution of influenza viruses.

## Results

### Inferring reassortment events between HA and NA genes

We reconstructed individual phylogenetic trees for each of the two surface proteins HA and NA for the two major influenza subtypes circulating in humans, H3N2 and H1N1. As expected, HA and NA phylogenies of the same subtype were incongruent. Using software GIRAF [32], we identified taxa that descended from within-subtype reassortant ancestors and thus inferred the positions of reassortment events on the phylogenies of an individual segment (see Materials and Methods for details). We inferred a total of 15 reassortment events between these two segments in subtype H3N2, and 5 events in subtype H1N1. We found that 847 out of 1,376 H3N2 isolates and 201 out of 745 H1N1 isolates are descendants of at least one reassortment event, which is consistent with previous findings [29,33,34]. To completely resolve incongruences between individual segment phylogenies, we assumed that reassortments are the only source of true differences between the phylogenies of individual segments. This assumption imposes a constraint that phylogenies of different segments may differ by at most as many rooted subtree prune-regraft (rSPR) operations as there are reassortment events, and otherwise be identical. We reconstructed such “constrained” phylogenies of individual segments using previously inferred “unconstrained” individual segment phylogenies as templates (see Materials and Methods for details).

### Identifying pairs of sites involved in positive inter-gene epistasis

**Fig 1:**
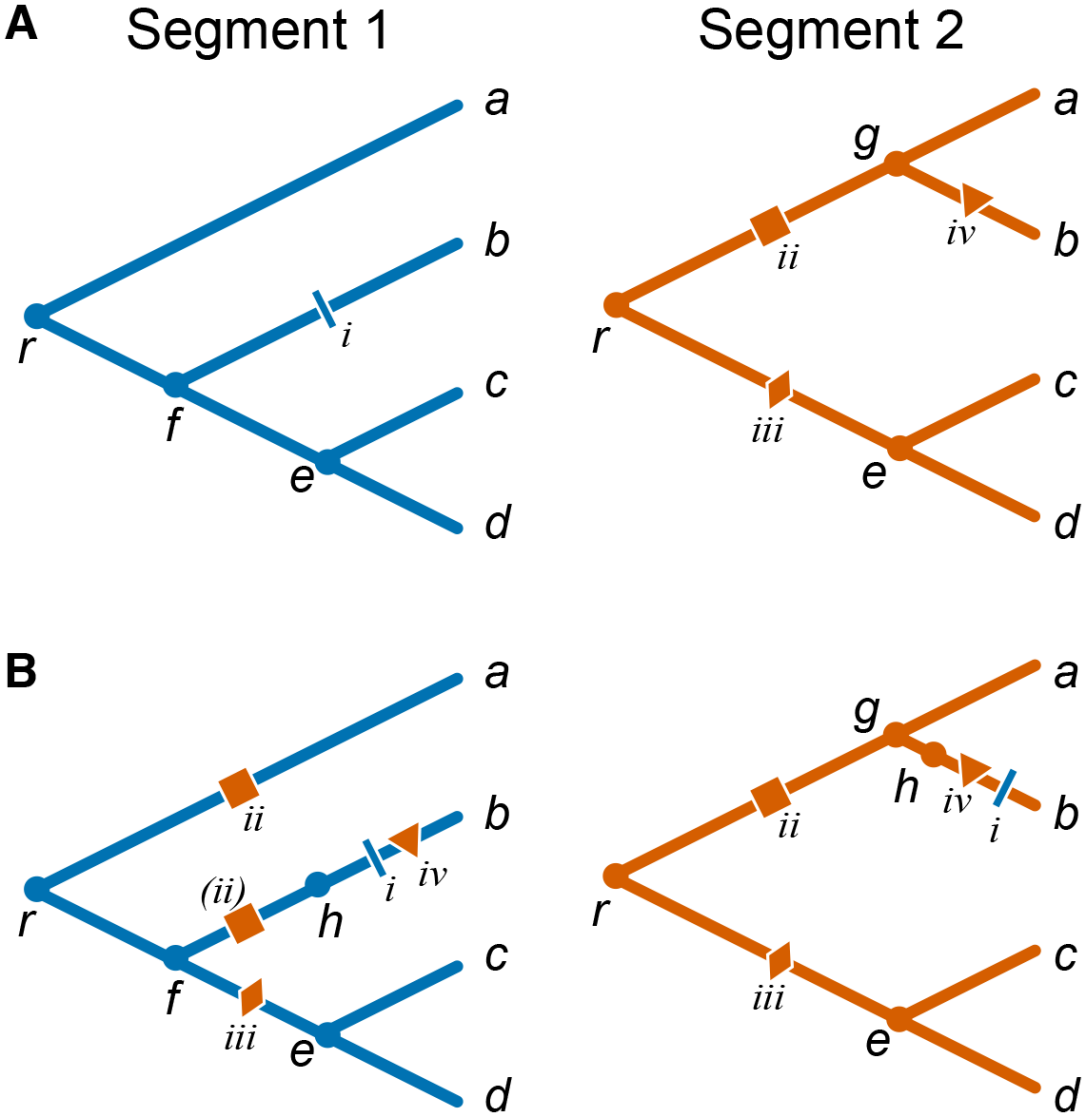
Mapping mutations between segments in the presence of reassortments. **(A)** Individual toy phylogenies for segments 1 (left) and 2 (right) with respective mutations. **(B)** Segment 1 phylogeny with segment 2 “background” mutations mapped onto it (left) and segment 2 phylogeny with segment 1 “background” mutations mapped onto it (right). *a–d*, leaf nodes; *e–g*, internal nodes; *h*, virtual node arising due to the reassortment event; *r*, root node; nodes corresponding to the two segments of the same isolate are denoted with the same letter. Each mutation is identified by a roman numeral and a unique symbol colored according to the segment in which it occurred. Mutation *ii* in segment 2 maps onto two branches of the segment 1 phylogeny, once as a regular mutation onto branch *ra* and once as a virtual mutation (denoted by parentheses) onto branch *fh*.

Accelerated origination of mutations at one site (referred to as “trailing” site) following a genetic change at another site (referred to as “leading” site) indicates that mutations at the trailing site are more beneficial after a mutation occurs at the leading site, and thus indicate positive epistasis [21,30]. Here we are specifically interested in situations when leading and trailing sites are located in different genes and therefore have potentially different evolutionary histories. This fact complicates the inference of the temporal order of mutations. Consider mutations *i* and *ii* in the toy example presented in Fig 1A. While both of them obviously occurred on the line of descent of isolate *b*, it is not immediately clear whether mutation *i* in segment 1 occurred before or after mutation *ii* in segment 2. We therefore cannot say a priori whether mutation *ii* facilitated mutation *i*, or mutation *i* facilitated mutation *ii*, or there was no interaction between them at all.

To resolve such ambiguities, we estimate the temporal order of mutations in different genes using the constrained phylogenies constructed above. Specifically, in order to study accelerated origination of mutations in one gene (referred to as the “foreground” gene) that follow mutations in the other gene (referred to as the “background” gene), we map all mutations in the background gene onto the phylogeny of the foreground gene. Since the constrained phylogenies are topologically identical with the exception of a relatively small number of reassortment events, most branches of the background tree correspond to unique branches of the foreground tree. Most mutations in the background gene are therefore unambiguously mapped onto branches of the foreground-gene phylogeny. In the toy example shown in Fig 1 branches *gb*, *ec*, and *ed* in the segment 2 phylogeny correspond to branches *fb*, *ec*, *ed* of the segment 1 phylogeny, respectively. Therefore, when considering segment 2 as the background gene, mutation *iv* unambiguously occurs on branch *fb* of the segment 1 phylogeny (Fig 1B).

Ambiguities in mapping background-gene mutations onto the foreground-gene phylogeny arise at branches that precede and follow reassortment events, such as branches *rg*, *ga*, and *re* in the segment 2 phylogeny in Fig 1A. For instance, mutation *iii* in segment 2 could occur either on branch *rf* or on branch *fe* of the segment 1 phylogeny. We resolve such ambiguities by placing background mutations onto the distal branch of the foreground phylogeny (e.g., in Fig 1, mutation *iii* is placed on branch *fe*). This choice minimizes the potential number of mutation pairs that contribute to our epistasis statistic (see below and Materials and Methods).

Finally, reassortment events themselves represent genetic changes in the background gene which may potentially elicit epistatic responses in the foreground gene. Indeed, when viewed as an event on the line of descent of a foreground-gene isolate, each reassortment event is a replacement of the genetic background gene, equivalent to gain of multiple simultaneous mutations which we call “virtual”. To account for the possibility that some of such virtual mutations in the background gene lead to acceleration in rates of origination of mutations at foreground-gene sites, we mark each reassortment events by a “virtual” node on the foreground-gene phylogeny. All foreground-gene mutations that occur on the respective branch are then placed after the virtual node. Here we make a simplifying assumption that reassortment events precede all mutations on the respective branch (see Materials and Methods for details). Even though this assumption introduces an error in our inference of relative order of mutations, this error is small because the fraction of branches involved in reassortment events is small. To illustrate this procedure, consider again the toy example in Fig 1. When considering segment 1 as the foreground gene, we posit that the reassortment (virtual node *h*) precedes mutation *i* on branch *fb* (segment 1) and mutation *iv* on branch *gb* (segment 2) which is also mapped onto branch *fb* of the segment 1 phylogeny (Fig 1B). This reassortment event replaces background segment 2 variant that carries no mutations (present at node *f*) with a segment 2 variant descendent from node *g* that has mutation *ii*. Thus, mutation *ii* is a virtual mutation in the background gene, and is placed on the virtual branch *fh*. Note that mutation *ii* is also mapped onto branch *ra* of segment 1 phylogeny. An alternative approach where we do not introduce the virtual node but assume a random order of all mutations (including virtual ones) within an edge yields qualitatively similar results (see Materials and Methods).

Once all genetic changes in the background gene are mapped onto the foreground-gene phylogeny, we can use our previously developed method [21] for detecting acceleration in the rate at which mutations arise on our phylogeny at sites in the foreground gene following mutations in the background gene. To do so, we compute the epistasis statistic for each pair of sites *(i*, *j)* where the leading site *i* is in the background gene and the trailing site *j* is in the foreground gene (see Materials and Methods). The epistasis statistic tends to be large for those pairs of sites in which a mutation at the trailing site quickly follows a mutation at the leading site and for which such mutations at the trailing site occur in multiple descendant lineages. We measure time between the leading and the trailing mutation as the number of synonymous mutations in the foreground gene that occur between them. As in our previous study [21], we exclude all mutations at terminal branches because many such mutations are likely to be deleterious or spurious.

Finally, to identify the pairs of sites with the epistasis statistic greater than expected by chance (which we call “putatively epistatic pairs”), we randomly reshuffle foreground-gene mutations among branches of the foreground-gene phylogeny while keeping the mapped background-gene mutations fixed. This permutation procedure preserves the number of mutations on each branch and the number of mutations observed at each sites, but breaks all potential associations between background- and foreground-gene mutations. It produces the null distributions of the epistasis statistic for all pairs of sites simultaneously and allows us to estimate the false discovery rate (FDR) for the number of putatively epistatic pairs at a desired nominal *P*-value threshold [21]. Importantly, our procedure does not take into account linkage between sites or temporal variation in the external selection pressure, which can inflate the epistasis statistic for some pairs and will lead to an underestimate of FDR. Thus, our list of putatively epistatis pairs of sites will likely contain some pairs that do not actually interact but have a significantly elevated value of the epistasis statistic for other reasons, e.g., hitchhiking. We discuss this important caveat in section “Confounding effect of hitchhiking on inference of epistasis” below and provide an estimate of the fraction of truly interacting pairs in our list.

### Prevalence of inter-gene epistasis in influenza surface proteins

**Fig 2:**
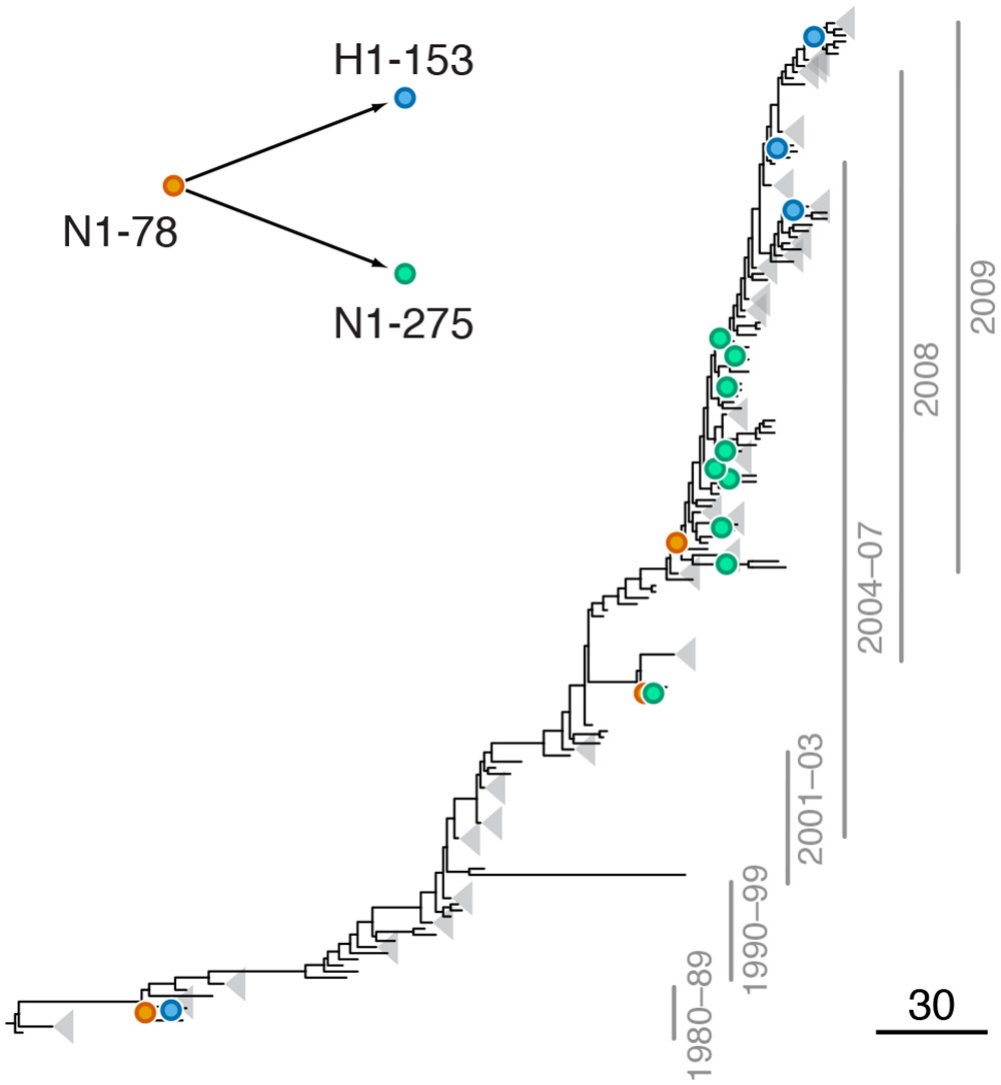
Example of putative inter-gene epistasis between sites N1-78 and H1-153. Mutations at sites N1-78 and H1-153 are marked by orange and blue circles, respectively; mutations at site N1-275 are marked by green circles. Site N1-275 was found to form a highly scoring intra-gene epistatic pair with site N1-78 in our previous study [21] (see text for details). Only mutations that form consecutive pairs are shown (see Materials and Methods); see S1 Data for all mutations. Vertical bars show years in which the isolates where sampled. The inset shows the inferred directionality of epistatic interactions with arrows pointing from the leading to the trailing sites.

**Fig 3:**
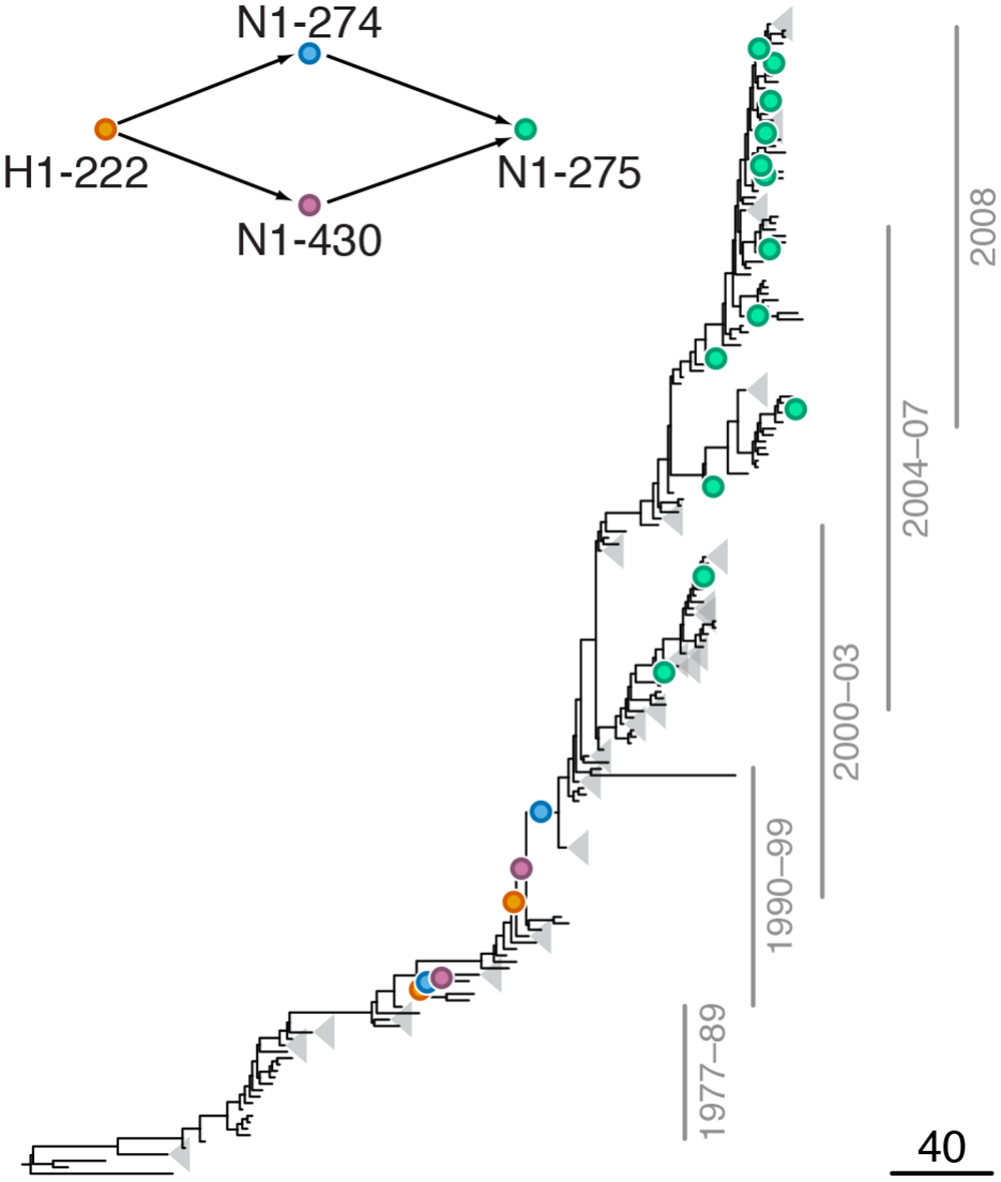
Example of putative inter-gene epistasis between sites H1-222 and N1-274. Mutations at sites H1-222 and N1-274 are marked by orange and blue circles, respectively; mutations at site N1-275 are marked by green circles. Site N1-275 was found to form a highly scoring inter-gene epistatic pair with site N1-274 in our previous study [21] (see text for details). Only mutations that form consecutive pairs are shown (see Materials and Methods); see S2 Data for all mutations. Vertical bars show years in which the isolates where sampled. The inset shows the inferred directionality of epistatic interactions with arrows pointing from the leading to the trailing site.

**Fig 4:**
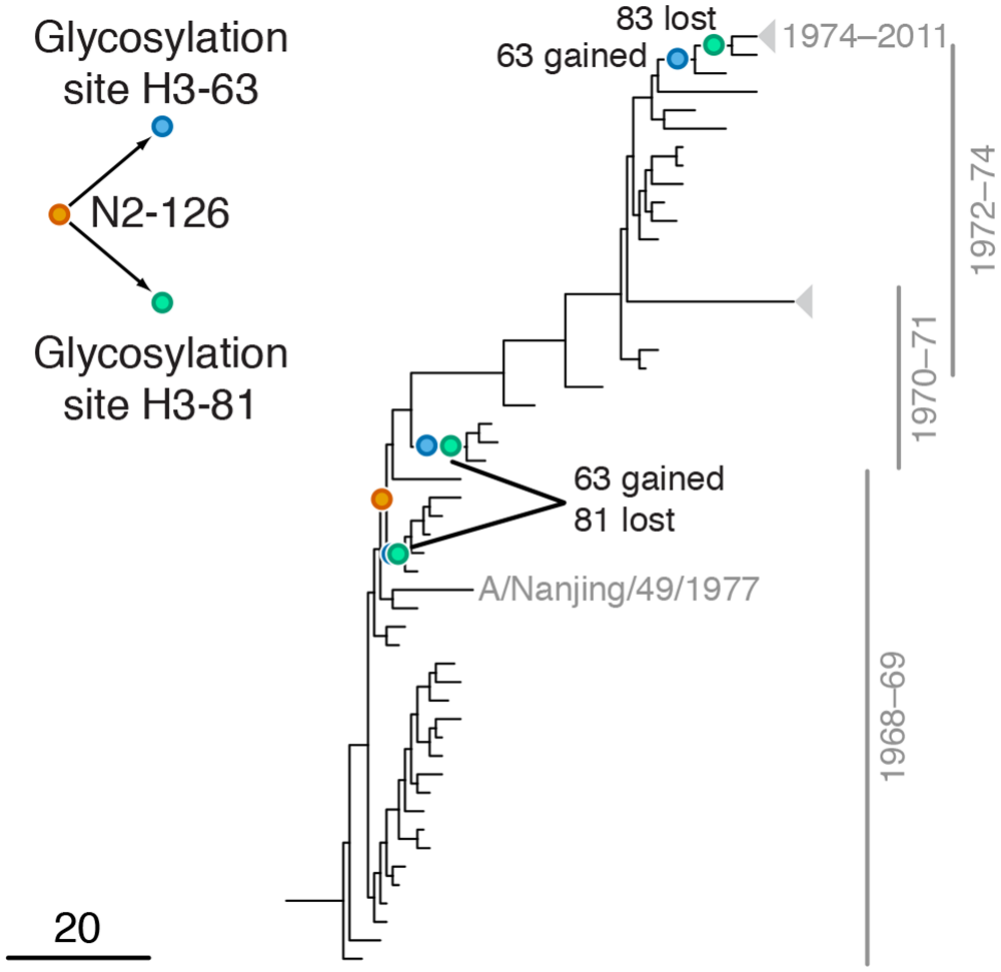
Example of putative inter-gene epistasis between sites N2-126 and H3-63 and H3-81. Mutations at sites N2-126 and glycosylation motifs starting at sites H3-63 and H3-81 are marked by orange, blue and green circles, respectively. All three mutations affecting the H3-63 glycosylation motif occurred at site H3-63 and created a new glycosylation site. Two mutations affecting the H3-81 glycosylation motif occurred at site H3-81 and one occurred at site H3-83, but all of them destroyed an existing glycosylation motif. Only mutations that form consecutive pairs are shown (see Materials and Methods); see S3 Data for all mutations. Vertical bars show years in which the isolates where sampled. The inset shows the inferred directionality of epistatic interactions with arrows pointing from the leading to the trailing site.

We considered both HA and NA as foreground and background genes, for both subtypes. In all cases, we found a higher than expected number of nonsynonymous site pairs with high values of the epistasis statistic in our data, implying abundant positive inter-gene epistasis between amino acid-changing mutations (Tables1 and S3, Figs 2–4 and S1). The observed number of epistatic pairs was significantly greater than expected for all considered nominal *P*-value thresholds below 0.05 in three of the four comparisons: (N2, H3), (H3, N2), and (H1, N1). Here and hereafter, the first segment listed in a pair is the background, and the second segment is the foreground. In the fourth comparison (N1, H1), it was significant for nominal *P*-values of 0.005 and below (S1 Fig). Hereafter, we refer to these thresholds as “liberal *P*-value thresholds”. To form conservative lists of putatively epistatic pairs of nonsynonymous sites, we chose the threshold nominal *P*-values that minimize the FDR, while still retaining enough sites for the downstream analyses (Table 1, see Materials and Methods; hereafter, “conservative *P*-value thresholds”). At conservative thresholds, the number of epistatically interacting pairs of nonsynonymous sites is about 5 times greater than expected by chance in three of the four comparisons: (N2, H3), (H3, N2), and (H1, N1), and about 2.5 times greater than expected in the remaining comparison (N1, H1) (Table 1; S1 Fig).

**Table 1:** Pairs of sites in HA and NA evolving under positive inter-gene epistasis.

Subtype	H3N2		H1N1	
Number of sequences	1,376		745	
Gene pair (background, foreground)	(N2,H3)	(H3,N2)	(N1,H1)	(H1,N1)
Foreground protein sites, total	563	459	566	470
Foreground protein sites, variable*	173	147	130	122
Total number of site pairs	25,431	25,431	15,860	15,860
Timescale parameter τ**	62	50	62	52
“Conservative” nominal *P*-value threshold	5×10^−4^	5×10^−4^	0.001	0.001
Significant pairs (expected)	5.92	4.78	5.76	5.18
Significant pairs (observed)	29	24	13	25
FDR, %	20	20	44	21
Distinct leading sites	23	21	13	25
Distinct trailing sites	13	11	5	6

*Number of sites variable on internal branches

**See Materials and Methods and Ref. [21]

At conservative *P*-value thresholds, between 11% and 19% of nonsynonymous sites were involved in epistasis as leading, and between 4% and 8%, as trailing, depending on the considered pair of genes. For example, among the variable nonsynonymous sites in H3, 8% (13/173) were involved as trailing sites in epistasis with N2, and 12% (21/173) were involved as leading sites (Table 1). Overall, between 20% (53/261 for N1) and 31% (123/392 for N2) of all observed nonsynonymous mutations occurred at sites that we classify as epistatically interacting (either leading, trailing, or both). The mean time between putatively epistatic leading and trailing mutations in different genes was about 5 years (S2 Fig), similarly to our finding for intra-gene epistasis [21].

### Confounding effect of hitchhiking on inference of epistasis

Evolution in large populations with limited recombination (such as influenza A) proceeds via selective sweeps whereby neutral and deleterious “hitchhiker” mutations linked to one or multiple advantageous “driver” mutations proceed to fixation all together [35–40]. Linkage may confound inferences of epistasis from phylogenetic patterns of mutations. For example, imagine that mutations at multiple sites sweep to fixation together but only one of these mutations facilitates a rapid spread of subsequent mutations at a trailing site. On the resulting genealogy, all mutations that participate in the sweep will form consecutive pairs with the trailing mutations, which will elevate the epistasis statistic for all such site pairs. Since our permutation procedure does not account for linkage, it may call all of these pairs as putatively epistatic. Thus, we expect a certain number of putatively epistatic pairs to be hitchhiking-induced false positives. In this section we show that this effect indeed takes place. We also show that it cannot account for all of the signal of epistasis that we see, and we provide a conservative estimate for the fraction of truly epistatic site pairs among all putatively epistatic pairs.

To show that hitchhiking confounds our ability to detect epistasis, we repeated our analyses using synonymous mutations in the background gene as leading and synonymous or nonsynonymous mutations in the foreground gene as trailing (syn-syn or syn-nsyn pairs, respectively). We found many putatively epistatic site pairs among syn-nsyn mutations (S3 Fig). Since synonymous mutations have little or no effect on protein structure, we expect that there would be few (if any) real epistatic interactions between synonymous mutations in one gene and non-synonymous mutations in another gene. (However, true syn-nsyn epistasis may potentially arise from viral RNA-protein interactions during packaging.) Synonymous substitutions nonetheless participate in selective sweeps that also involve non-synonymous mutations [38], some of which may experience epistatic interactions. Thus, significant values of the epistasis statistic among syn-nsyn pairs most likely arise as a result of hitchhiking. To confirm this, we found that for most of putatively epistatic syn-nsyn pairs, there exists a putatively epistatic pair of non-synonymous sites (nsyn-nsyn pair) with the same trailing mutations and a leading site whose phylogenetic distribution of non-synonymous mutations is identical to that of the leading synonymous mutations in the syn-nsyn pair. Such cases comprise 53% of all syn-nsyn pairs for (N1,H1), 64% for (H1,N1), 74% for (N2,H3), and 67% for (H3,N2) for the liberal *P*-value thresholds. As expected, the fraction of syn-nsyn site pairs that have a corresponding nonsyn-nonsyn pair with an identical phylogenetic distribution of mutations is lower for those syn-nsyn pairs that are formed by multiple leading mutations (e.g., 36% for (H3,N2)), compared with pairs formed by just one leading mutation (e.g., 75% for (H3,N2)). Finally, as expected, the signal of epistasis among syn-syn pairs was very weak or non-existent (S4 Fig). The small residual signal may still be attributed to hitchhiking or to a small number of as yet unexplained real genetic interactions.

Overall, our method does not reliably identify which of the leading mutations that co-occur on the same edges of the phylogeny actually precipitate subsequent epistatic trailing mutations. However, we can provide a conservative estimate for the number of truly epistatic pairs among all putatively epistatic pairs. To do this, we grouped together all nsyn-nsyn pairs with identical phylogenetic distributions of leading and trailing mutations and ordered them according to the lowest *P*-value in such “phylogenetic group” (S3 Table). Each group with a low group *P*-value signifies that the trailing mutations raise in frequency together unexpectedly rapidly after a previous selective sweep that involves the leading mutations. This implies that at least one of the leading sites exhibits positive epistasis with at least one of the trailing sites. An average phylogenetic group involves 1.54 site pairs for (N1,H1), 1.85 for (H1,N1), 1.59 for (H3,N2), and 1.64 for (N2,H3). Thus, assuming that each group includes only one truly epistatic pair, we estimate that the fractions of truly epistatic site pairs constitute 65% for (N1,H1), 54% for (H1,N1), 63% for (H3,N2), and 61% for (N2,H3); the actual fractions may be even higher, as there may be multiple drivers with identical phylogenetic distributions [38,41].

**Fig 5:**
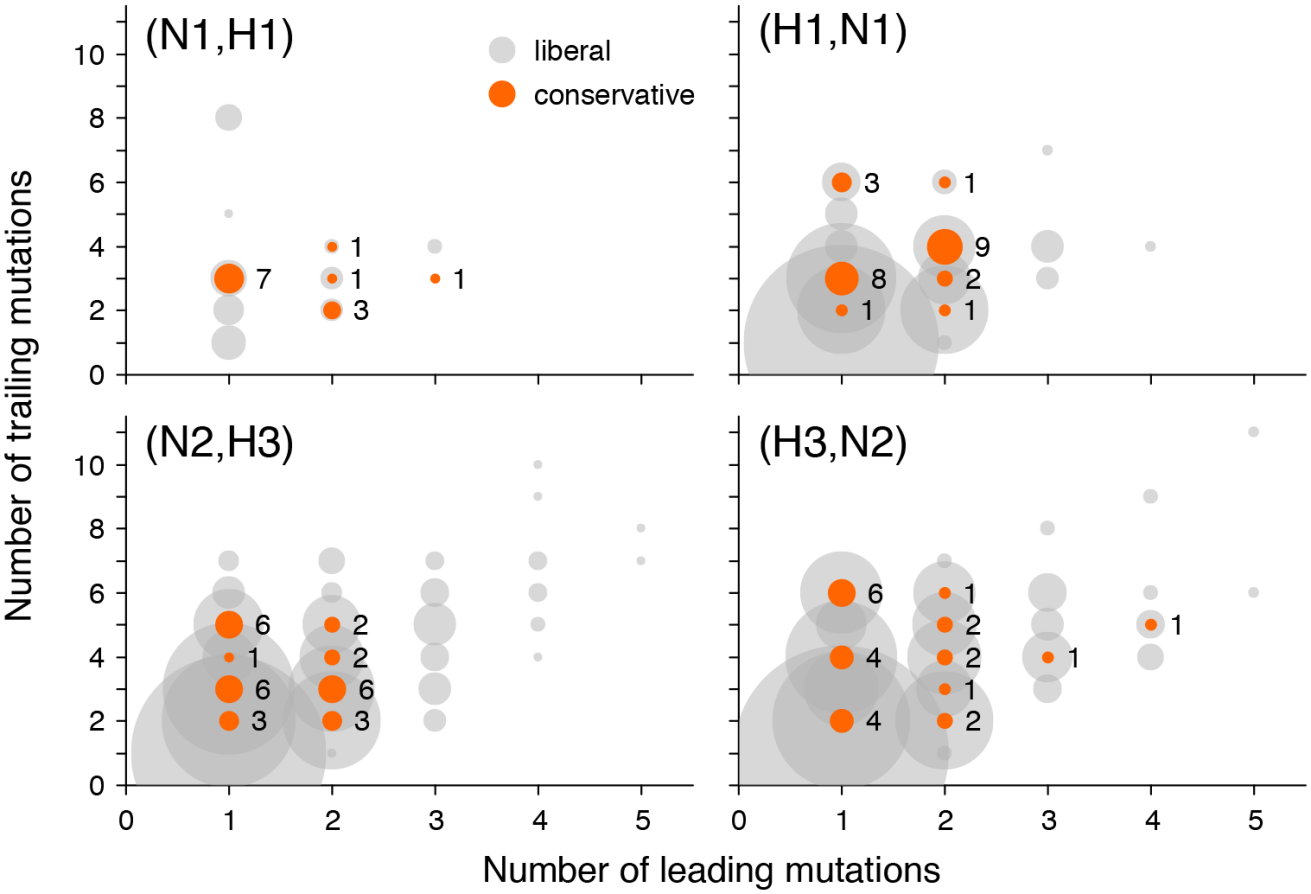
Numbers of leading and trailing mutations at putatively epistatic site pairs in different gene pairs. Circle area corresponds to the number of site pairs with each number of leading and trailing mutations at liberal (grey) or conservative (orange) *P-*value thresholds. To gauge the scale, the numbers of pairs at conservative *P-*value thresholds are shown next to the orange circles.

As an additional evidence supporting our claim that not all putatively epistatic pairs are results of hitchhiking, we repeated our analysis for a restricted subset of site pairs where a mutation at the trailing site follows a mutation at the leading site in at least two independent locations on the genealogy (Fig 5). In this smaller subset of data, we still observe more site pairs with high values of the epistasis statistic than expected, and this excess is statistically significant (S5 Fig, S3 Table). Putatively epistatic site pairs revealed in this analysis must have a smaller fraction of hitchhiking-induced pairs because the same non-interacting mutations will only rarely follow each other closely in two or more distinct sweeps.

### Reassortments and inter-gene epistasis

The direct effect of reassortments on our results was moderate: between 67% and 91% of pairs of nonsynonymous mutations at putatively epistatic site pairs were not separated by any reassortment events (S1 Table). In over 75% of all putatively epistatic site pairs, the majority of consecutive mutations did not span any reassortment events.

To analyze the association between reassortments and inter-gene epistasis, we asked whether putatively epistatic pairs are formed more frequently than expected with leading mutations that arose during reassortments, i.e., mutations at virtual nodes. We found that for 6% (N2, H3), 20% (H3, N2), 0% (N1, H1) and 10% (H1, N1) of trailing mutations at putatively epistatic pairs of sites the corresponding leading mutation was at a virtual node (S1 Table). This is more than expected (10%) for N2 (binomial *P*-value = 4 × 10^−4^), less than expected (19%) for H3 (binomial *P-*value = 2 × 10^−4^), and not significantly different from what is expected for N1 and H1. Therefore, in N2, a substantial number of trailing mutations compensates for the changes in the genetic background brought about by reassortment events.

### Inter-gene versus intra-gene epistasis

How does the extent of inter-gene epistasis compare to the extent of intra-gene epistasis? To address this question, we repeated the analyses of intra-gene epistasis from Ref. [21] with the data for each of the four genes analyzed here (H1, N1, H3, and N2) and compared the number of putatively epistatic inter- and intra-gene site pairs for each FDR value (S6 Fig). We found that the number of intra- and inter-gene putatively epistatic pairs is comparable when trailing mutations occur in HA and leading mutations occur in HA or NA, respectively (S5 Fig). At the same time, the number of putatively epistatic inter-gene pairs exceeds that of intra-gene pairs by as much as a factor of 3 when trailing mutations occur in NA (S6 Fig).

We also compared the sets of sites involved in inter-gene epistasis to sets of sites involved in intra-gene epistasis [21]. The overlap between these two groups of sites was slightly higher than expected by chance for the H3 leading and the N2 trailing sites in the (H3,N2) gene pair (Table 2), but this difference was not significant after Bonferroni correction.

**Table 2:** Comparisons of sets of sites evolving under inter-gene vs. intra-gene epistasis.

	Inter-gene epistasis		Intra-gene epistasis [21]		Enrichment or depletion	
Gene	Site type	Count	Site type	Count	*P*_+_	*P*_–_
H3	**leading**	**21**	**leading**	**50**	**0.040**	**0.960**
	trailing	13	trailing	79	0.070	0.928
N2	leading	23	leading	35	0.651	0.354
	**trailing**	**11**	**trailing**	**58**	**0.013**	**0.985**
H1	leading	25	leading	54	0.187	0.813
	trailing	5	trailing	66	0.731	0.267
N1	leading	13	leading	39	0.075	0.925
	trailing	6	trailing	57	0.651	0.349

For HA and NA proteins, the subsets of leading and trailing sites in inter-gene epistatic pairs (subset 1) were compared with the subsets of leading and trailing sites in intra-gene epistatic pairs (subset 2). Significantly (*P*<0.05) enriched categories (*P*_+_) are in boldface, and depleted (*P*_–_), in italic.

### Enrichment of functional sites among epistatic sites

Next, we investigated whether sites that were implicated in inter-gene epistasis occurred preferentially in parts of the HA and NA proteins with known functional significance. In particular, we compared the sets of putatively epistatic sites with the sets of epitopic sites [20,42–46], glycosylation sites [47,48], sites that are responsible for antigenic cluster transitions [36,49–51], as well as sites that evolve under uniform or lineage-specific positive selection.

The sites in HA identified as interacting with NA occurred in all parts of HA protein, with the majority of them located in known antigenic epitopes. This is expected because our method has more power to identify epistasis at sites that are more variable, and most of variable sites are also epitopic. However, we can control for this bias by comparing the set of putatively epistatic sites that we discover in real datasets with the corresponding sets of sites discovered in permuted datasets (see Materials and Methods). Using this approach, we find that leading sites in HA are actually not enriched for epitopic sites or sites under uniform or lineage-specific positive selection (Table 3). But they are enriched for sites responsible for differences between antigenic clusters [36,49] (Table 3). On the other hand, cluster-transition sites are slightly underrepresented among trailing sites in H3. Finally, glycosylation sites [52] are underrepresented among trailing sites in both H3 and H1 (Table 3).

**Table 3:** Comparisons of sets of sites evolving under inter-gene epistasis with sets of sites with known properties.

	Inter-gene epistasis		Category			Enrichment or depletion	
Gene	Site type	Count	Site type	Reference	Count	*P*_+_	*P*_–_
H3-HA1	leading	19	**antigenic**	[36]	**44**	**0.023**	**0.975**
			**antigenic**	[49]	**49**	**0.035**	**0.965**
			antigenic	[50]	7	0.255	0.745
			epitopic	[42,43]	131	0.128	0.873
			glycosylation	[47]	7	0.253	0.748
	trailing	9	*antigenic*	[36]	*44*	0.993	0.005
			*antigenic*	[49]	*49*	1.000	0.000
			*antigenic*	[50]	*7*	0.990	0.008
			epitopic	[42,43]	131	0.670	0.333
			*glycosylation*	[47]	*7*	0.978	0.020
H3-HA0	leading	21	uniform positive^a^	-	17	0.355	0.648
			lineage positive^b^	-	27	0.470	0.535
	trailing	13	uniform positive^a^	-	17	0.945	0.053
			lineage positive^b^	-	27	0.908	0.090
N2	leading	23	pitopic	[43–45]	45	0.620	0.378
			uniform positive^a^	-	10	0.593	0.405
			lineage positive^b^	-	16	0.405	0.595
	trailing	11	epitopic	[43–45]	45	0.158	0.840
			uniform positive^a^	-	10	0.558	0.440
			lineage positive^b^	-	16	0.910	0.088
H1-HA1	leading	21	antigenic	[51]	41	0.160	0.838
			epitopic	[20]	32	0.133	0.865
			glycosylation	[48]	11	0.528	0.470
	trailing	5	antigenic	[51]	41	0.708	0.295
			epitopic	[20]	32	0.138	0.863
			*glycosylation*	[48]	*11*	0.958	0.040
H1-HA0	leading	25	uniform positive^a^	-	6	0.588	0.415
			lineage positive^b^	-	12	0.515	0.483
	trailing	5	uniform positive^a^	-	6	0.318	0.680
			lineage positive^b^	-	12	0.258	0.740
N1	leading	13	epitopic	[46]	12	0.138	0.863
			*uniform positive* ^*a*^	*-*	*2*	0.993	0.005
			lineage positive^b^	-	6	0.945	0.053
			glycosylation	[48]	13	0.713	0.285
	trailing	6	epitopic	[46]	12	0.160	0.838
			uniform positive^a^	-	2	0.253	0.745
			lineage positive^b^	-	6	0.193	0.808
			glycosylation	[48]	13	0.130	0.868

For HA and NA proteins, the subsets of leading and trailing sites in inter-gene epistatic pairs (subset 1) were compared with the subsets of leading and trailing sites in a range of other categories (subset 2). Significantly (*P*<0.05) enriched categories (*P*_+_) are in boldface, and depleted (*P*_–_), in italic.

^a^Sites under uniform positive selection (HyPhy model IFEL).

^b^Sites under lineage-specific positive selection (HyPhy model MEME).

We found no enrichment of epitopic, positively selected, or glycosylation sites among sites in NA that are involved in inter-gene epistasis (Table 3). To better understand what types of sites in NA comprise the putatively epistatic set, we searched the literature for evidence of functional consequences of mutations at sites that we identified. (Such a systematic analysis was impractical for HA because much less site-specific functional data is available for this protein.) We carried out this search for each of the 31 distinct sites in N2 (23 leading, 11 trailing sites, and 3 sites falling into both types), and for each of the 19 epistatic sites in N1 (13 leading and 6 trailing).

We found that inter-gene epistasis in both N1 and N2 may be related to NA catalytic activity and resistance to inhibitors (S2 Table). Specifically, we found that N2 sites 370, 372, 401 and 432, which are homologous to the second sialic-binding site (hemadsorption site) of avian influenza viruses, are the trailing sites in 58% (14/24) of the discovered epistatic pairs, including 13 pairs with the lowest *P*-values, and that they are leading sites in 10% (3/29) of the discovered epistatic pairs (S2 Table). Although the function of these sites in human influenza has not been directly demonstrated, it is thought that they affect catalytic efficiency of NA [53]. Among the remaining epistatic sites, leading sites 126 and 248 and trailing site 127 affect binding of NA inhibitors. In addition, two more leading sites, 215 and 332, although not shown to affect NA activity, were reported to often mutate in response to NA inhibitor treatment. Finally, two leading sites, 172 and 399, and one trailing site 263 were previously inferred to distinguish the reassortant H3N2 clades [33].

In N1, three leading sites 59, 386 and 388 (70, 390, and 392 in N2 numbering), and trailing site 434, undergo host-specific position-specific glycosylation, likely affecting enzymatic activity of NA [54]. Leading sites 6 and 14 and trailing site 15 are located in the transmembrane domain, which affects viral sialidase activity through its effect on NA tetramer assembly and transport to the membrane [55,56]. Leading site 149 and trailing sites 83, 275, 267 and 287 (274, 266, and 286 in N2 numbering) affect sialic acid binding; mutations at sites 267 and 275 were also shown to affect resistance to oseltamivir, including the mutation at site 275 which gave rise to the common oseltamivir-resistant H1N1 subtype. Finally, mutations at leading site 78, and trailing site 83, arise with H275Y in naturally oseltamivir-resistant strains.

### Examples of biologically plausible explanations for epistasis

Finally, we present several examples of implicated epistatic site pairs with biologically plausible explanations for the mechanism of their epistatic interactions.

#### Leading site 78 in N1, trailing site 153 in H1

We found a strong signal of epistasis between sites N1-78 and H1-153 (site 156 in H3 numbering; S3 Table, Fig 2). Site N1-78 was also implicated in an intra-gene epistatic interaction with site N1-275 (site 274 in N2 numbering) [21]; mutation at site N1-275 causes oseltamivir resistance [52]. Mutations at site N1-78 predated H275Y mutations at least twice (Fig 2). Importantly, mutations at site 153 in H1 are known to be responsible for changes in receptor binding affinity [20], suggesting that a single mutation (N1-78) may precipitate further functionally important mutations in multiple genes.

#### Leading site 222 in H1, trailing sites 274 and 430 in N1

We found a strong signal of epistatic interactions between site H1-222 (site 225 in H3 numbering) and sites N1-274 and N1-430 (S3 Table, Fig 3), both of which, in turn, were implicated in intra-gene epistasis with site N1-275 [21]. Interestingly, mutations at site N1-430 modify activity of NA [53], while mutations at site H1-222, which is part of the receptor binding site, have been shown to compensate mutations in NA that confer resistance to NAI [54]. Since resistance to NAI depends on the balance between catalytic activities of HA and NA [23,55], mutations at these sites may be important for maintaining this balance.

#### Leading site 126 in N2, trailing sites 63 and 81 in H3

We found a strong signal of epistasis between leading site N2-126 and trailing sites H3-63 and H3-81 (Fig 4). Site N2-126 frequently mutates in MDCK lines [56], and the observed mutation H126P is implicated to be important for the avian to human host shift of the H3N2 subtype [57]. Sites H3-63 and H3-81 are parts of known glycosylation motifs [47,58]. The loss of glycosylation site at position 81 in 1974 follows the gain of glycosylation site at position 63 in 1973 soon after beginning of H3N2 pandemic in 1968 [58], possibly in response to the H126P mutation in N2. Mutations at site H3-63 in three independent lineages created a new glycosylation site, while the old glycosylation site was concordantly lost either via a mutation at site H3-81 (twice) or a mutation at site H3-83 (once; Fig 4). Glycosylation of HA often masks epitopes [58,59] and loss of glycosylation at site 81 may also affect receptor binding [60]. We speculate that adaptation to the new host occurred via a change in receptor-binding activity of NA that in turn precipitated compensatory mutations in HA glycosylation patterns.

## Discussion

We developed a phylogeny-based method for detecting positive epistasis between mutations at sites that are incompletely linked. This approach provides the first systematic procedure for identifying such genetic interactions from sequence data sampled over time. We demonstrated the power of this method by applying it to data from human influenza A virus where we found dozens of putative epistatic interactions between sites in the surface proteins HA and NA. Our analysis cannot take the place of a direct experimental assay to unambiguously demonstrate epistasis between a specific pair of mutations in influenza viruses. Still, several of the most significant pairs of sites implicated by this statistical procedure have known biological functions that provide a plausible mechanistic basis for the observed patterns of coordinated molecular evolution.

While powerful, our method of detecting epistasis between incompletely linked sites has three limitations. First, it relies on our knowledge of recombination breakpoints and on our ability to accurately infer phylogenies, detect recombination events and map mutations from one phylogeny onto another. Since within-segment recombination in influenza is rare [31] and the main source of horizontal exchange of genetic material is reassortment, RNA segments represent well-defined linkage blocks, which simplifies our analysis. Although in principle our method of detecting epistasis through temporal clumping of mutations on the phylogeny should be applicable to systems with recombination (e.g., HIV), a practical implementation becomes cumbersome because recombination breakpoints need to be determined and mutations need to be mapped onto several different phylogenies. Even in influenza, an accurate detection of reassortments is difficult, especially between closely related taxa [29,32], and the mapping of mutations is inherently ambiguous.

The second limitation is inherent to the problem of detecting epistasis from temporal mutation data, and is discussed in detail in a previous study using such techniques [21]. The problem is that our method (as well as any method utilizing the same data) will identify sites as trailing in epistatic pairs if mutations at these sites are temporally clustered for any reason—including reasons that are not caused by epistatic interactions *per se*. In theory, temporally correlated substitutions may arise due to episodes of positive selection or relaxed negative selection that are correlated between sites of the two genes. If this scenario is common, we would typically expect the inverse of a site pair with a high epistatic score to also be high-scoring. However, among the pairs defined under the conservative *P-*value threshold, we do not observe a single such pair. While there were several site pairs with a significant inverse pair among the pairs defined under the liberal *P*-value threshold, these pairs do not have lower *P*-values than the remaining pairs, arguing against the temporally correlated selective constraint as the predominant explanation for our results. Furthermore, the sites detected by our method do not tend to experience lineage-specific positive selection (Table 3), again arguing against this explanation.

Finally, hitchhiking of mutations at linked sites is a major confounding factor for our method. This problem is relevant for human influenza A because hitchhiking is widespread in its evolution [35–39]. However, hitchhiking alone cannot account for all of the detected epistasis signal because our randomization procedure preserves the number of mutations at each branch, and thus accounts for temporal non-uniformity of evolutionary rates. Nevertheless, the list of epistatic pairs is likely contaminated with hitchhikers that co-occur on branches together with the actual leading mutations [21]. Hitchhiking is apparently the leading cause of most of the epistatic signal that we observe between leading synonymous sites and trailing non-synonymous sites. By contrast, by grouping site pairs into phylogenetic groups with identical phylogenetic distributions of mutations, we estimate that less than 50% of putatively epistastic non-synonymous pairs arose due to hitchhiking. In each phylogenetic group, those site pairs with lower *P*-values and those that have multiple leading mutations are more likely to be the true epistatic pairs.

Keeping these caveats in mind, we turn to the interpretation of our observation of epistasis between mutations in the HA and NA. Between 20% and 31% of all mutations occurred at sites involved in epistatic interactions as either leading or trailing. The numbers of putatively epistatic site pairs where the leading mutation occurs in NA and the trailing mutation occurs in HA and vice versa are similar. The mean times between consecutive mutations at such sites are also similar between each other (S2 Fig) and to those in intra-gene epistasis [21]. Thus, both mutations in HA facilitated by prior mutations in NA and mutations in NA facilitated by prior mutations in HA appear to be common. The evolution of these two segments of the human influenza virus is therefore tightly coordinated. Moreover, trailing sites in NA more frequently follow a leading mutation in HA than a leading mutation in NA itself.

What is the molecular basis for such coordinated evolution? We searched for enrichment of various properties among epistatically interacting sites. In HA, we found no enrichment of epistatic sites among the positively selected sites. In fact, it is somewhat surprising that the detected putatively epistatic sites are not particularly rapidly evolving, despite the fact that our method has more power to detect epistasis at sites with more mutations [21]. We do observe, however, an enrichment of leading sites among the HA sites responsible for antigenic shifts. This suggests that the changes in HA driven by immune system pressure are frequently compensated by mutations in NA. Conversely, antigenic sites and glycosylation sites were underrepresented among the trailing sites of HA, suggesting that the HA trailing sites compensating for the mutations in NA comprise a novel potentially interesting set of functional sites in this protein.

We also found no enrichment of positional or functional categories in epistatic sites in NA. This lack of clear pattern is consistent with experimental data and implies that genetic interactions occur through a wide range of mechanisms, and that the sites involved in them are hard to predict a priori [60,61]. However, we observed that many epistatic sites in NA are involved in NAI resistance, modulation of NA activity, or both. Why do the sites affecting these traits interact with HA? Some of the observed interactions (e.g., site N1-78 (Fig 2), and sites N1-274 and N1-430 (Fig 3)) could be directly attributed to the requirement to balance the activities of HA and NA to maintain viral fitness, especially in the presence of NAI [55]. Other interactions may affect this balance indirectly. For example, sites in the signal peptide of HA appear to occasionally interact with sites in the transmembrane domain of NA, e.g., site H1-16 forms a putatively epistatic pair with site N1-15 (S3 Table). These types of mutations likely affect the efficiency of membrane localization of the respective surface proteins [62], and mutations in the transmembrane domain may also influence NA activity through their effect on tetramer assembly [63].

Some of the putatively epistatic site pairs that we detected have been experimentally confirmed. For example, a number of mutations in HA of H1N1 closely predated the 2007 spread of the H275Y (274 in N2 numbering) oseltamivir resistance mutations in NA. Recently, 7 of these HA sites were experimentally tested for interactions [61]. These experiments showed that HA that carries the derived residues at all seven sites is well adapted to both the ancestral H275 (sensitive) and the derived Y275 (resistant) variant of NA. At the same time, three out of seven reconstructed reversions in HA (at sites 82, 141 and 189) had large fitness defects in the context of the derived NA variant, implying that mutations at these sites compensated for the H275Y mutation in NA [61]. Remarkably, all three of these HA sites form high-ranking pairs in our analysis with the site 275 in NA (S3 Table, sites 99, 157, 205 in our numbering). Finally, a recent experimental study [27] confirmed the involvement of site 275 in intragenic epistatic interactions predicted in our previous work [21].

Trailing mutations in N2 frequently compensate for the changes in H3, and possibly in other genes [29], brought about by reassortments. Furthermore, the N2 sites that are involved in intra-gene epistasis as trailing are enriched in sites that experience post-reassortment mutations [29], and in sites involved in inter-gene epistasis as trailing (Table 2). These findings support our interpretation that NA is the gene most actively involved as trailing in epistatic interactions, with mutations at it compensating a range of other events both in the same [21] and in other genes (Table 2; [29]); and show that the same set of sites in N2 might tune the protein function in response to various changes of genetic background of H3N2 IAV.

More generally, our results suggest that the evolution of a protein depends strongly on its genomic context, with a substantial number of adaptive mutations representing responses to mutations that previously occurred in other proteins. Such evolutionary coupling between different proteins has also been observed in several experimental systems [13,15,23,64–66]. However, estimating the fraction of mutations that are driven by direct adaptation to the external environment versus by selection to balance or compensate the effects of prior mutations elsewhere in the genome remains an important open problem.

## Materials and Methods

### Sequences

We downloaded all complete human H3N2 influenza A isolates (N = 2,205) available on 27 October 2011 and all complete human seasonal H1N1 influenza A isolates (N = 1,180) available on 12 November 2012 from the flu database [67]. The amino acid sequences were aligned using MUSCLE [68,69], and the alignments were reverse translated using PAL2NAL [70]. Genotypes containing truncated sequences or long stretches of unidentified nucleotides were discarded. The 3 genotypes of H3N2 subtype carrying indels were discarded. We also discarded all genotypes of H1N1 that were sampled prior to 1936 because they had large (15–16 amino acids) gaps between amino acid positions 42 and 77 in the NA protein. In all sequences, the alignment columns with gaps in more than 10% of all sequences were excluded from further consideration; in the remaining alignment columns, gaps were substituted with the consensus nucleotide.

Four isolates of H1N1 subtype (A/New Jersey/1976, A/Wisconsin/301/1976, A/Iowa/CEID23/2005, A/Switzerland/5165/2010) were discarded as swine-origin influenza virus (SOIV) [71–73]. Three isolates of H3N2 subtype (A/Ontario/RV123/2005, A/Ontario/1252/2007 and A/Indiana/08/2011) were discarded as SOIV triple reassortants [74].

Many of the genotypes had NA genes with identical nucleotide sequences; among each such set of genotypes, we only retained one random genotype. This reduced our sample to 1,376 isolates for H3N2 subtype, and 745 isolates for H1N1 subtypes.

For HA and NA proteins of H1N1, the numbering scheme used through the text is relative to the proteins of the A/AA/Huston/1945 isolate, unless stated otherwise.

### Inferring the temporal order of mutations in two reassorting segments

We asked whether a mutation at a particular site in HA segment facilitates a subsequent mutation at a particular site in NA segment, or vice versa. To address this, we need to reconstruct the phylogenetic trees for each of the two segments, infer the position of reassortments on these trees, and establish the temporal order of mutations in different segments relative to each other. We achieve this goal in three steps, which are described in detail below. Briefly, in the first step, based on topological incongruencies between the phylogenetic trees of individual segments, we identify the so-called reassortment sets, i.e., sets of taxa that are likely descendants of reassortant viruses. In the second step, we reconstruct the so-called constrained phylogenies of the segments, i.e., phylogenies that are topologically identical everywhere except for branches that correspond to reassortment events. This allows us to map, in the third step, the mutations that occur on branches of one phylogeny to the branches of another phylogeny.

#### Inferring the reassortment and the “trunk” sets

We used GiRaF [32] to identify sets of taxa that are descendant to reassortment events. To reduce the computational burden associated with this step, we first clustered isolates with nucleotide identity exceeding 99.5% across the concatenated HA-NA sequence using CD-HIT [75], and retained for the GiRaF analysis one random sequence from each cluster, for a total of 225 H3N2 and 169 H1N1 isolates.

GiRaF takes as input the sets of phylogenetic trees sampled from their posterior distributions for each segment. We obtained 1000 such trees per segment using MrBayes [76] with the GTR+I+Γ model, 2 million iterations, sampling one tree every 2000 iterations. The output of GiRaF is a collection of taxon sets each of which consists of descendants of a likely reassortment event. Because GiRaF attempts to infer nested reassortments and because of phylogenetic noise, these sets are generally overlapping, i.e., the same taxon may be included into multiple sets. However, to infer subtrees with topologies unaffected by reassortments, we need non-overlapping sets of taxa each descendant to the same past reassortment event (or the same series of such events). To construct such non-overlapping sets, we sorted the GiRaF sets according to the fraction of taxa shared with other sets, from high to low. All taxa in the highest-ranking set were then considered as one set of reassortants. We then excluded these taxa from all lower-ranking sets, resorted the remaining GiRaF sets, and repeated the procedure. Thus, for example, if GiRaF set 1 was fully nested within a larger GiRaF set 2, we inferred two non-overlapping sets of reassortants: those of set 1, and those of set 2 excluding those of set 1. A GiRaF set not overlapping any other GiRaF sets always produced a set of reassortants of its own. By this procedure, each taxon was included either into a unique reassortment set (denoted by the most recent reassortment event), or into the set of non-reassortant taxa which we refer to as the “trunk” set. We then ascribed the isolates removed in the clustering step to the same set as their representative cluster sequence.

#### Reconstructing constrained phylogenies

Given *N* sets of taxa (*N*–1 reassortment sets and one trunk set), we reconstruct two complete phylogenies (one per segment) that differ by exactly *N*–1 rooted subtree prune-regraft (rSPR) operations corresponding to *N*–1 reassortment events. We call such phylogenies “constrained”. To assemble constrained phylogenies, we start by reconstructing two standard maximum likelihood phylogenies (one per segment) using PhyML [77] (model GTR+I+Γ) and rooting these phylogenies with the oldest isolate as the outgroup (A/Albany/18/1968 for H3N2, and A/Henry/1936 for H1N1). We use these phylogenies as templates for reconstructing constrained phylogenies.

Next, for each reassortment or trunk set of taxa, we reconstruct an unrooted phylogenetic subtree from the alignment of concatenated HA and NA sequences by maximum likelihood using PhyML [77] (model GTR+I+Γ). To root each such subtree, we compare the locations of the most recent common ancestors (MRCAs) of this set of taxa on two template trees. In the absence of phylogenetic noise, MRCAs on both segments would be identical, in which case the root of the concatenate-based subtree would be placed unambiguously. However, in general, MRCAs based on different template phylogenies are different. We therefore place the root of the concatenate-based subtree in such a way that its position is most similar to both alternative MRCA positions according to a trade-off function described in S1 Text. As a result of this procedure, we obtain *N*–1 reassortment rooted subtrees and one trunk rooted subtree.

We then assemble these subtrees into two complete constrained phylogenies (one per segment) that differ by exactly *N*–1 rSPR operations as follows. In the absence of noise, i.e., if reassortments were the only source of differences between the two template phylogenies, each reassortment set would be either mono- or paraphyletic on each template phylogeny. Each reassortment subtree could then be unambiguously grafted into a unique branch of another (paraphyletic) subtree, in exact accordance with the template tree. However, some reassortment sets are polyphyletic on the template trees, making the grafting procedure ambiguous. Our algorithm resolves such ambiguities on the basis of a tradeoff between two criteria: maximizing topological similarity between the constrained and the template phylogeny for each segment, and minimizing the length of the resulting constrained phylogeny (see S1 Text for details).

The result of this assembly is a pair of phylogenetic trees, one tree per segment, that differ from each other by *N–*1 rSPR operations, as desired. Once the topologies of the constrained phylogenies are reconstructed, we optimize their branch lengths and infer ancestral sequences using HyPhy with the nucleotide REV+Rate Het. model [78].

#### Establishing temporal order of events on the phylogeny

Our goal is to detect mutations in one segment that occurred after mutations in another segment. If we analyze mutations in segment 1 that occurred after mutations in segment 2, we say that “segment 2 forms the genetic background for segment 1”, or that “segment 2 is in the background” and “segment 1 is in the foreground”.

To study such mutations in segment 1, we map mutations in the background segment (segment 2) onto the foreground-segment phylogeny. Since the topologies of each reassortment and trunk subtree are identical for both segments by construction (see above), each branch of the background tree maps to a unique branch of the foreground tree (we call such branches “unambiguous”), with the exception of branches that are involved in the rSPR operations (we call such branches “ambiguous”). Thus, mutations in the background segment that occur on unambiguous branches map onto unique branches of the foreground phylogeny. Consider a toy example shown in Fig 1. Node *b* forms a single reassortment set, and the remaining nodes form the trunk set. Correspondingly, the constrained phylogenies shown for the two segments differ by a single rSPR operation involving the branch leading to node *b*. Therefore, branches *gb*, *ec*, and *ed* in the segment 2 phylogeny map to branches *fb*, *ec*, *ed* of the segment 1 phylogeny, respectively, and mutation *iv* in segment 2 unambiguously occurs on branch *fb* of the segment 1 phylogeny (Fig 1B).

Now consider ambiguous branches, such as branches *rg*, *ga*, and *re* in the segment 2 phylogeny in Fig 1A. Each rSPR corresponding to each reassortment event removes one node (prune operation), thus merging a pair of successive branches, and adds one node (regraft operation), thus splitting a branch. Therefore, each reassortment event results in one branch of the background phylogeny corresponding to a pair of branches of the foreground phylogeny (1-to-2 map) and another pair of branches of the background phylogeny corresponding to another branch of the foreground phylogeny (2-to-1 map). In Fig 1, a pair of branches *rg* and *ga* of the segment 2 phylogeny corresponds to branch *ra* of the segment 1 phylogeny, and branch *re* of the segment 2 phylogeny corresponds to the pair of branches *rf* and *fe* of the segment 1 phylogeny. In 2-to-1 maps, all mutations that occur on either of the two branches in the background segment are unambiguously mapped onto a single branch of the foreground segment phylogeny. In Fig 1, mutation *ii* in segment 2 maps unambiguously onto branch *ra* of the segment 1 phylogeny. The situation is more difficult in the 1-to-2 maps, where each mutation that occurs on such ambiguous branch in the background segment could map onto either one of the two branches in the foreground segment. In Fig 1, mutation *iii* in segment 2 could occur either on branch *rf* or on branch *fe* of the segment 1 phylogeny. We resolve this ambiguity by placing all such background mutations onto the distal branch of the foreground phylogeny (e.g., in Fig 1, mutation *iii* is placed on branch *fe*). This choice minimizes the number of consecutive potentially epistatic mutation pairs that can form between the background and the foreground sites (see below).

Finally, each reassortment event maps onto the branch of the foreground segment phylogeny that leads to the most recent common ancestor of the corresponding reassortment subtree. We refer to such branches as “reassortment-carrying branches”, or RCBs. We signify the occurrence of a reassortment event by adding a “virtual” node on the RCB and placing all foreground mutations that occur on the RCB after the virtual node. Thus, we make a simplifying assumption that the reassortment event precedes all mutations on the RCB. For example, in Fig 1B, reassortment (virtual node *h*) precedes mutations *i* and *iv* on branch *fb* of segment 1 phylogeny. (Alternative approach of randomly shuffling all mutations, including virtual ones, within the edge yielded a highly similar rank order of epistasis statistic *P*-values (Spearman’s rho > 0.98).) When viewed as an event on the foreground phylogeny, each reassortment event is equivalent to an instantaneous replacement of the background segment sequence present in the parent node of the foreground RCB with the background segment sequence present at the parental node of corresponding RCB on the background segment phylogeny. In the example shown in Fig 1B, the reassortment event replaces segment 2 which carries no mutations (present at node *f*) with a segment 2 descendent from node *g* that has mutation *ii*. Thus, this reassortment event is equivalent to the occurrence of mutation *ii* in the background segment. This “virtual” mutation is placed on the virtual branch *fh*. This procedure of mapping background mutation onto the foreground phylogeny guarantees that the order of background mutations is preserved.

All procedures for construction of constrained gene trees were implemented in C++ with bio++ package [79,80]. Mapping of mutations was implemented in Perl and used Bio::Phylo package [81].

### Inferring positive epistasis

#### Epistasis statistic

To infer positive epistasis between mutations at two sites mapped onto the same phylogeny, we employ the method previously described in [21]. Briefly, for each pair of sites (*i*,*j*), we first identify the set *S*_*ij*_ of all consecutive mutations pairs, i.e. such mutation pairs where a mutation at site *i* is on the line of descent of a mutation at site *j* with no other mutations at either site occurring in between. We then compute the epistasis statistic for this pair which in the simplest case is given by

$$E_{\tau }(i,j)=\sum_{\pi \in S_{ij}}\text{exp}\left\{-t_{\pi }/\tau \right\},$$

where the summation is taken over all consecutive mutation pairs, *t*_π_ is the time (measured in synonymous mutations) between the mutations in the pair π, and τ is the time-scale parameter which we choose to be equal to the average time 〈*t*_*π*_〉 averaged over all mutations at all site pairs (Table 1). For a more general expression of the epistasis statistic see [21]. The epistasis statistic for site pair (*i*,*j*) is large when (a) the set of consecutive mutation pairs is large and (b) when the mutations at the trailing site quickly follow the mutations at the leading site. Thus, site pairs with an unusually high epistasis statistic likely evolve under positive epistasis. Nonsynonymous leading and nonsynonymous trailing, synonymous leading and nonsynonymous trailing, and synonymous leading and synonymous trailing pairs of mutations are considered separately.

#### Identifying epistatic site pairs and computing the false discovery rate (FDR)

In order to identify site pairs with unusually high values of the epistasis statistic, we obtained the null distribution for the epistasis statistic at all site pairs simultaneously by randomly reshuffling mutations at all sites in the foreground gene among all branches of the phylogeny while keeping the mapped background mutations in place. This is more conservative than permuting both background and foreground mutations because this procedure preserves more features of the data. This permutation conserves the number of mutations at each site and on each branch thus controlling for possible biases introduced by differences in variability among sites and by the heterogeneity of mutations on the phylogeny [21]. We carried out 10,000 permutations for each analysis. Using the resulting null distributions for each site pair, we obtained the list of site pairs that were significant at any given nominal *P*-value (observed positives, OP). To estimate the number of false positives (FP) that we expect to find at a given nominal *P*-value, we selected 400 out of 10,000 permutations as fake datasets and calculated the number of significant site pairs in each of these fake datasets at that *P*-value. Thus, we obtained the null distribution of the number of significant pairs for each nominal *P*-value, which allowed us to estimate the expected number of FP (EFP) under the null hypothesis as well as the *P*-value for the number of OP. The FDR is then given by the ratio EFP/OP.

To test the robustness of our method with respect to hitchhiking, we carried out the same analysis as described above but restricted to those pairs of sites that had two or more non-virtual leading mutations. The fact that mutations are non-virtual guarantees that they arose as independent events rather than by a single event and subsequent reassortment. In particular, we first found those pairs of sites that exhibited two or more non-virtual leading mutations in the real dataset (real total, RT) as well as in each of 400 fake datasets (fake total, FT). Note that because the number of such site pairs differs from on datasets to another, we could no longer meaningfully compare the absolute number of observed positives (OP) with the expected absolute number of positives (EFP) among such pairs. Instead we obtained the *P*-value for the observed fraction of positives (OP/RT) by comparing it with the distribution of the fraction of positives (FP/FT) in the fake datasets.

### Testing for functional enrichment among sites involved in inter-gene epistasis

Lists of epitopic sites of HA were taken from [42,43] for H3N2 and from [20] for H1N1. Lists of epitopic sites of NA were taken from [43–45] for H3N2, and from [46] for H1N1. Sites involved in intra-gene epistasis in HA and NA of H3N2 and H1N1 were taken from [21]. Sites that may carry mutations changing the antigenic properties of isolates were taken from [36,49,50] for H3, and from [51] for H1. Glycosylation sites were taken from [47] for H3-HA1, and from [48] for H1-HA1 and H1-NA. Sites in H1, N1, H3 and N2 under uniform positive selection were inferred by HyPhy IFEL method [82], and under lineage-specific selection, by HyPhy MEME method [83] (*P*-value < 0.05) using Datamonkey web service [84,85] (http://www.datamonkey.org).

To test whether a particular set of sites *S* is enriched or depleted among the top-ranking epistatic leading sites compared to the random expectation, we used the following procedure. First, we defined for each site its leading *P*-value as the lowest nominal *P*-value among all site pairs with this site as leading. We then defined the leading test statistic as the difference between the medians of site’s leading *P*-value for sites in *S* and for sites not in *S*. The null distribution of this test statistic was determined from the 400 fake datasets generated from the no-epistasis null hypothesis as explained above. An analogous procedure was used to find enrichment among the top-ranking epistatic trailing sites.

For each pair of genes, we considered “leading-trailing” pairs of mutations to be provisionally “reassortment-induced” if the leading mutation was virtual, and the trailing mutation occurred in the same reassortment set. To test whether the reassortment-induced pairs are overrepresented, we analyzed the fraction of such mutations among all pairs of mutations at epistatic site pairs in the same reassortment set. The expected fraction was obtained from our ‘fake’ datasets (see above). We tested the null hypothesis that the observed number of reassortment-induced pairs of mutations was sampled from a binomial distribution with the probability of success equal to the expected fraction of such mutation pairs.

## Supporting Information

S1 Text Building constrained phylogenies. (DOC)

S1 Table Distribution of locations of consecutive mutation pairs relative to reassortment events. (XLS)

S2 Table Properties of epistatic sites in N1 and N2 neuraminidases. (XLS)

S3 Table Putatively epistatic site pairs at liberal *P*-value thresholds. “Phylogenetic group” refers to a group of site pairs where the leading and the trailing mutations occurred on the same sets of tree branches. Site pairs formed by more than one leading mutation are in bold. The last 12 columns identify the location of consecutive mutations relative to reassortment events: the first letter denotes whether the leading and the trailing mutations are located on the same (S) or different (D) reassortment subtrees; the second and the third letters denote the branch types on which the leading and the trailing mutations are located: R = reassortment-carrying branch; V = virtual branch; I = any other internal branch. Sheet 1: (N2,H3). Sheet 2: (H3,N2). Sheet 3: (N1,H1). Sheet 4: (H1,N1).
(XLS)

S1 Fig The number of observed (orange line) and expected (black line) epistatic nsyn-nsyn pairs significant at each nominal *P*-value threshold in different gene pairs. In each gene-pair annotation, the first gene refers to the background and the second gene refers to the foreground. Small (large) circles denote cases when the observed number exceeds the expectation according to the permutation test at significance level 0.05 (0.01). Arrows show the “conservative” *P-*value thresholds (see Results and Materials and Methods for details). In the (N1,H1) analysis we found no significant pairs at nominal *P*-value thresholds below 0.001.
(PDF)

S2 Fig The distribution of average times (in years) between putatively epistatic leading and trailing nonsynonymous mutations in different gene pairs. (PDF)

S3 Fig The number of observed (blue line) and expected (black line) epistatic syn-nsyn pairs at each nominal *P*-value threshold in different gene pairs. Notations as in S1 Fig.
(PDF)

S4 Fig The number of observed (blue line) and expected (black line) epistatic syn-syn pairs at each nominal *P*-value threshold in different gene pairs. Notations as in S1 Fig.
(PDF)

S5 Fig The fraction of observed (orange line) and expected (black line) epistatic nsyn-nsyn pairs among site pairs with at least two distinct leading mutations, significant at each nominal *P*-value threshold in different gene pairs. Notations as in S1 Fig.
(PDF)

S6 Fig The expected number of true positive epistatic pairs (which is given by OP–EFP, see Materials and Methods) in the inter-gene (orange line) and intra-gene (blue line) epistasis analysis, at each FDR. Notations as in S1 Fig.
(PDF)

S1 Data Phylogenetic tree in FigTree [86] format with all mutations at sites N1-78, N1-275, and H1-153 highlighted. (TRE)

S2 Data Phylogenetic tree in FigTree [86] format with all mutations at sites H1-222, N1-274, N1-430, and N1-275 highlighted. (TRE)

S3 Data Phylogenetic tree in FigTree [86] format with all mutations at sites N2-126, H3-63 and H3-81 highlighted. (TRE)
