# Supplementary material for: Coordinated Evolution of Influenza A Surface Proteins
Source: PLoS Genet. 2015 Aug 6;11(8):e1005404. doi: 10.1371/journal.pgen.1005404 (PMC4527594; doi:10.1371/journal.pgen.1005404)
Supplement: S1 Text — (DOC) [file pgen.1005404.s001.doc]

## Text S1

### Building constrained phylogenies

Subtree rooting. The subtree corresponding to the trunk subset is rooted using the outgroup sequence. Other subtrees are rooted based on the positions of the MRCAs of corresponding subsets of taxa on the two template trees. Generally, these positions don’t match on the two template trees. We then employ the following procedure to root the subtree. If at least on one of the template trees, the distance from the MRCA to its nearest daughter node *l* is nonzero, we root the subtree according to the template tree with the maximal *l* (assuming that longer branches correspond to more robust bipartitions). We then place the root of this subtree onto the branch with the maximal topological similarity score *s* (see below) to the bipartition that corresponds to the MRCA node of the subset of taxa on the template tree. Otherwise, we calculate *s* for each branch of the subtree with bipartitions by MRCA of the corresponding subset of taxa on each of the two template trees, and place the root on the branch with the maximal sum of the two scores.

Topological similarity score. To find a branch on the tree that corresponds to a particular bipartition, we calculate the topological similarity score *s* between this bipartition and the bipartitions defined by each branch of the tree, as described in Ref. [80]. *s* ranges between 0 and 1, so that *s*=1 only when the bipartition exactly matches a branch of the tree. The score between two bipartitions of unequal size is calculated only for those taxa that are present in both bipartitions.

Assembly of constrained topologies. Let V_0_ be the trunk set, and let V_i_, i = 1, 2, …, N–1 be the reassortment sets. Consider one of the two template trees, and let M_i_, i = 0, 1, …, N–1 be the MRCA node on this template tree for the corresponding set V_i_. The parent node of node M_i_ divides the template tree into three clades, A_i_, B_i_, and R_i_, such that A_i_ is the clade that contains all V_i_ taxa (and possibly other taxa), R_i_ is the clade that contains the root of the template tree, and B_i_ is the clade that contains all remaining taxa (empty for V_0_). In the absence of noise, all reassortment sets and the trunk set are mono- or paraphyletic on the template tree, implying that there is a unique “acceptor” set j_acc_ (i)  i whose members belong to both R_i_ and B_i_, but not to A_i_, and each of the remaining sets *j* ≠ *i* is entirely contained in one of the clades A_i_, R_i_, or B_i_. In this ideal case, subtree *i* should then be obviously grafted into the subtree j_acc_ (i). However, this is generally not the case. We therefore choose the acceptor subtree with the highest support for grafting on the template tree according to the following criterion. For each taxon set j, j = 0, 1,…,N–1, we define *V_j_^U^* to be the set of its members that also belong to a clade U*;* we identify the acceptor subtree j_acc_(i) for subtree *i* by sorting all taxon sets j by the size of their set $V_{j}^{R_{i}}\cup V_{j}^{B_{i}}$, from high to low, with all sets j for which $V_{j}^{A_{i}}=$ *Ø* preceding all those for which $V_{j}^{A_{i}}\neq$ *Ø*, and choosing the first set in this list.

Once the acceptor subtree is chosen, we graft subtree i onto a branch of the acceptor tree. The branch used for grafting is chosen so that it minimizes the distance between the graft *i* and the acceptor j_acc_ (i), preferably among the branches with high topological similarity to the acceptor branch on the template tree. Specifically, we choose the branch for grafting among the set of branches *V^h^* such that their topological similarity *s* to the bipartition of the corresponding acceptor subset on the template tree is above the threshold *h=*0.7. If no branches with *s>h* are found, we search for the optimal implantation point among all branches. The optimal branch connecting the graft subtree *i* and the acceptor subtree *j=* j_acc_ (i) is the branch that minimizes expression

$$\frac{1}{4}[L(V_{i}^{r_{i}},V_{j}^{p})+L(V_{i}^{r_{i}},U_{j}^{p})+L(U_{i}^{r_{i}},V_{j}^{p})+L(U_{i}^{r_{i}},U_{j}^{p})]-\frac{1}{2}[L(V_{i}^{r_{i}},U_{i}^{r_{i}})+L(V_{j}^{p},U_{j}^{p})],$$

where *r_i_* and *r_j_* are subtree roots; *p* is a bipartition of the acceptor subset; and *L(A,B)* is the mean distance between pairs of taxa within subsets *A* and *B.* As a result of this procedure, the subtree *i* is grafted into the subtree *j,* or forms a sister clade with it.

The order for the assembly of subsets is established from the template tree. For this, we build a matrix of phylogenetic distances (i.e., lengths of connecting paths along the phylogeny) between the subsets on the template tree. A distance between a graft and acceptor subsets is defined as the length of the branch connecting the acceptor point to the graft root. The trunk subset can be only an acceptor subset. The subtrees corresponding to the two subsets with the lowest distance between them in this matrix are joined together; a new matrix is then built, and the procedure repeated iteratively until all subtrees are assembled. There are three options for joining a pair of subtrees: either one can be grafted within the other, or they can form sister clades; to distinguish between these options, the same relationship is used as that in the template tree.
