# Supplementary figures and images for: Coordinated Evolution of Influenza A Surface Proteins
Source: PLoS Genet. 2015 Aug 6;11(8):e1005404. doi: 10.1371/journal.pgen.1005404 (PMC4527594; doi:10.1371/journal.pgen.1005404)

Number of positives

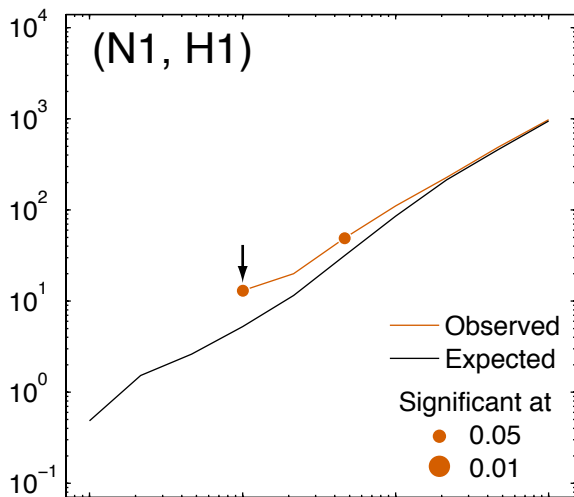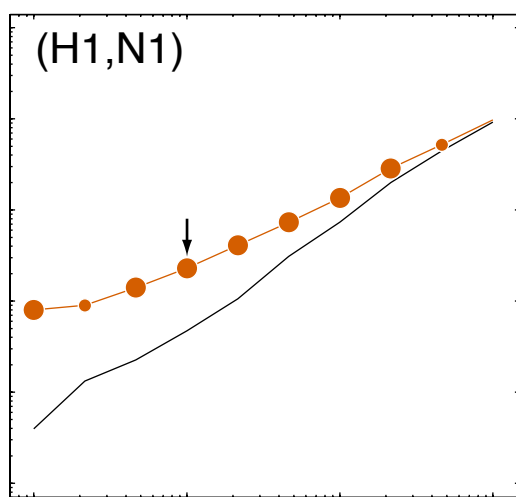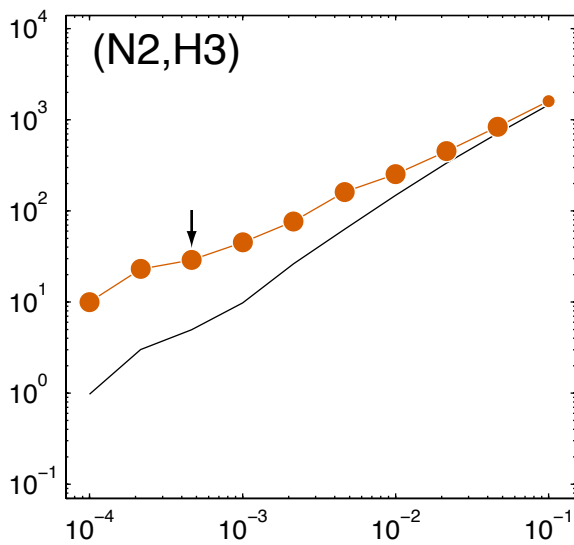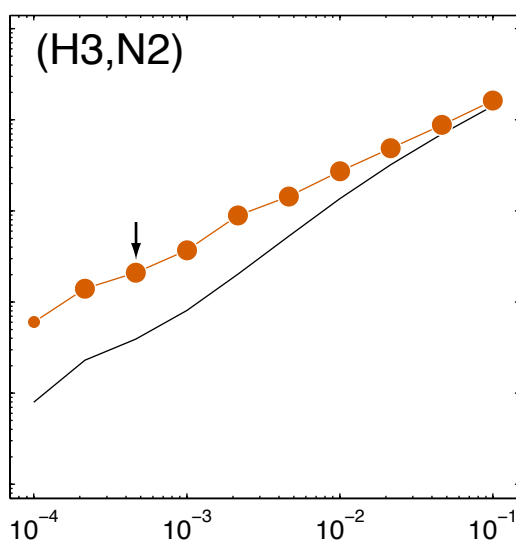

Nominal P-value

Supplement: S1 Fig — In each gene-pair annotation, the first gene refers to the background and the second gene refers to the foreground. Small (large) circles denote cases when the observed number exceeds the expectation according to the permutation test at significance level 0.05 (0.01). Arrows show the “conservative” P-value thresholds (see Results and Materials and Methods for details). In the (N1,H1) analysis we found no significant pairs at nominal P-value thresholds below 0.001. (PDF) [file pgen.1005404.s005.pdf]

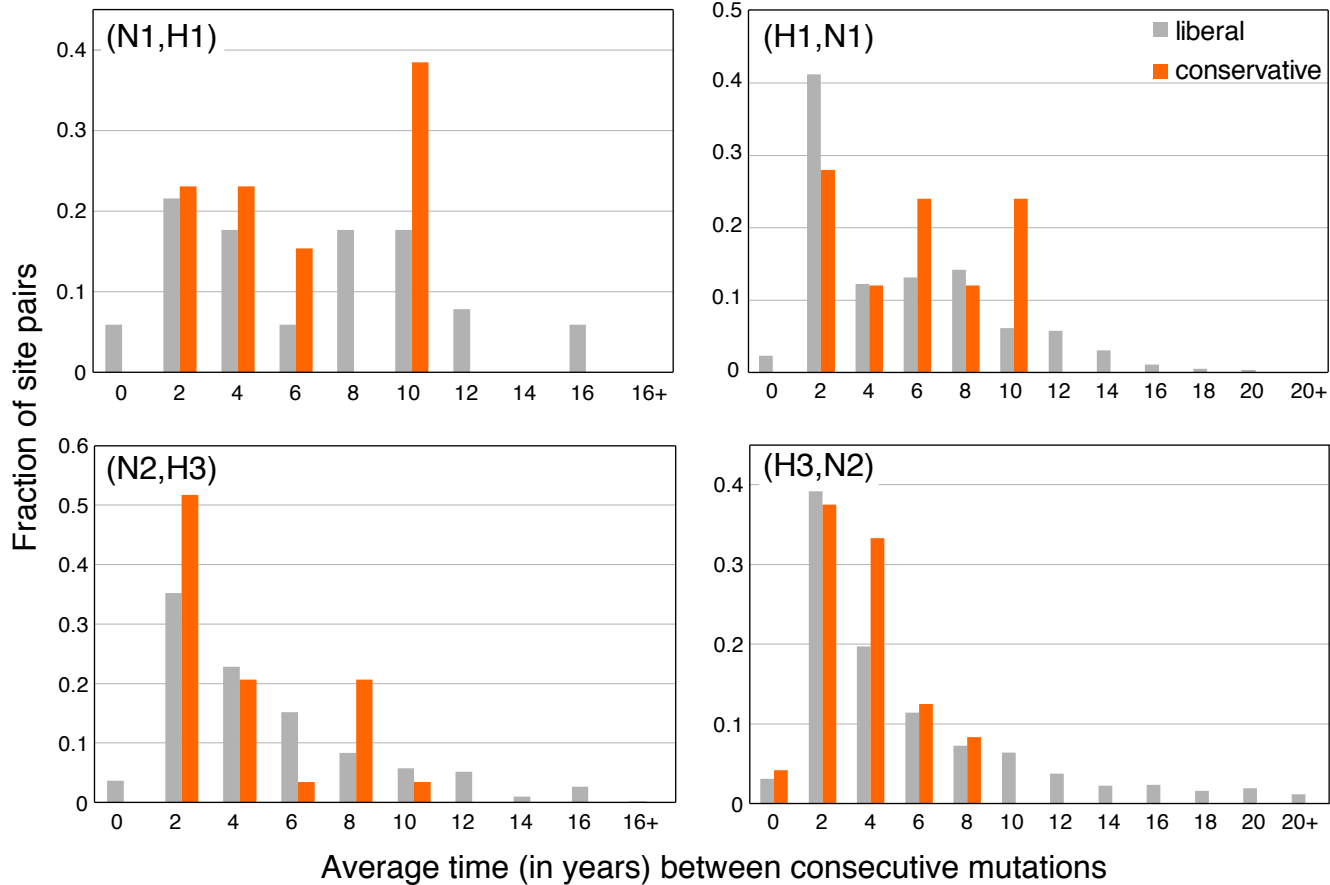

Supplement: S2 Fig — (PDF) [file pgen.1005404.s006.pdf]

Number of positives

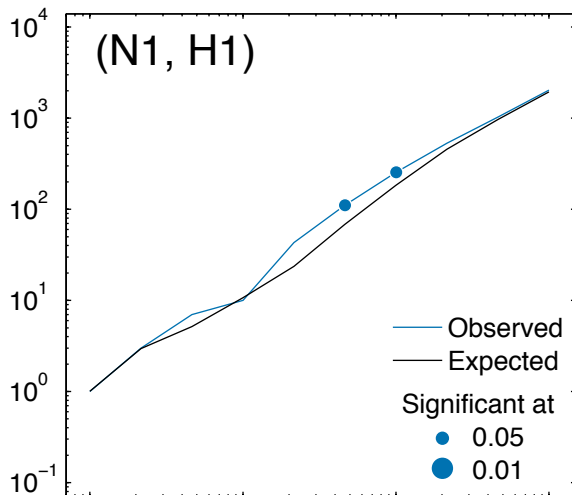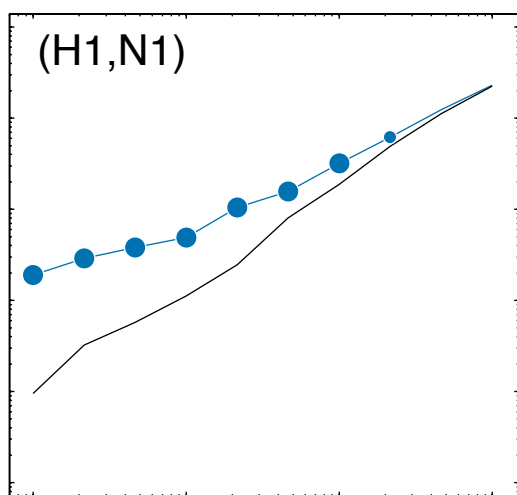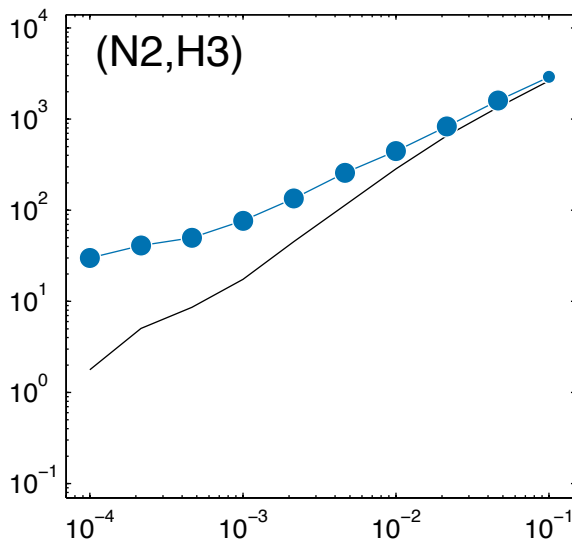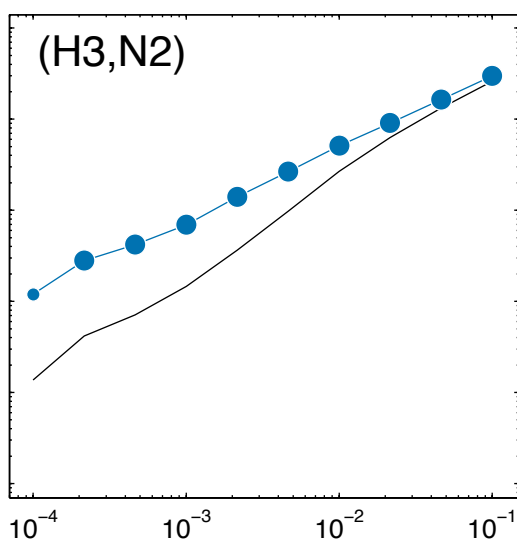

Nominal P-value

Supplement: S3 Fig — Notations as in S1 Fig. (PDF) [file pgen.1005404.s007.pdf]

Number of positives

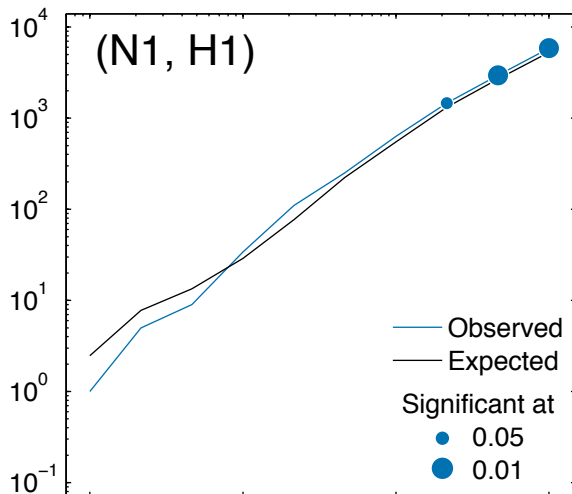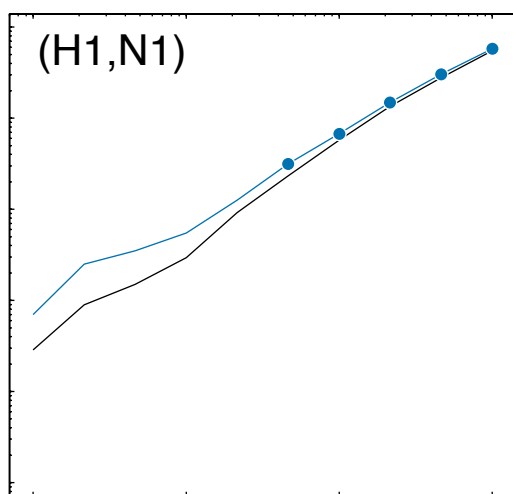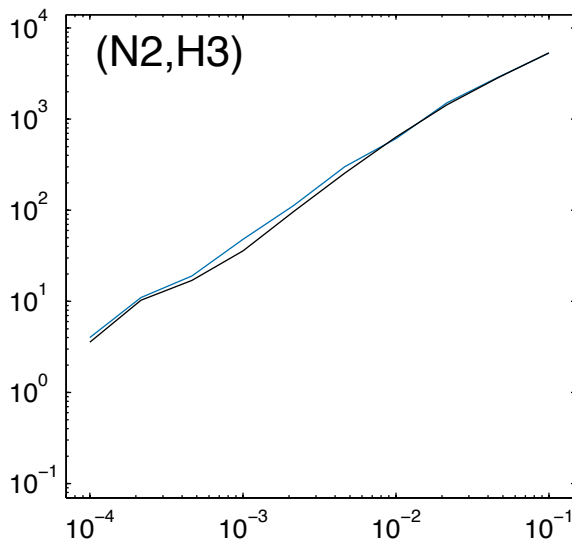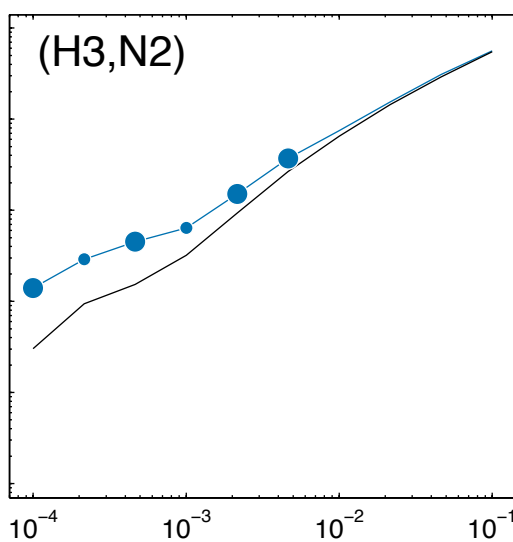

Nominal P-value

Supplement: S4 Fig — Notations as in S1 Fig. (PDF) [file pgen.1005404.s008.pdf]

Number of positives

(N1, H1)

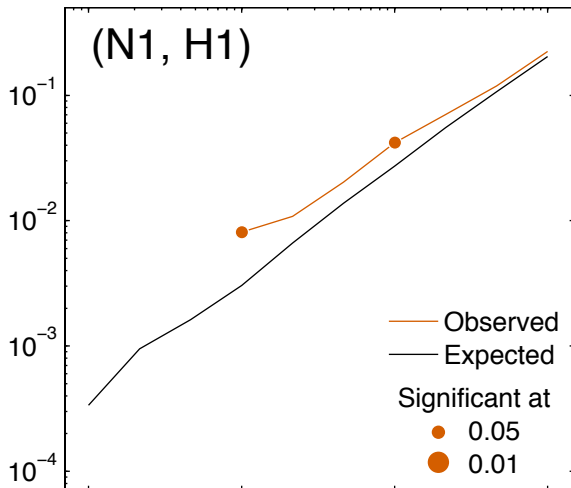

(H1,N1)

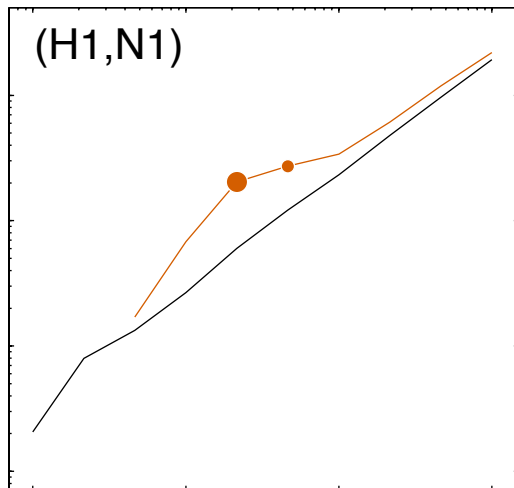

(N2,H3)

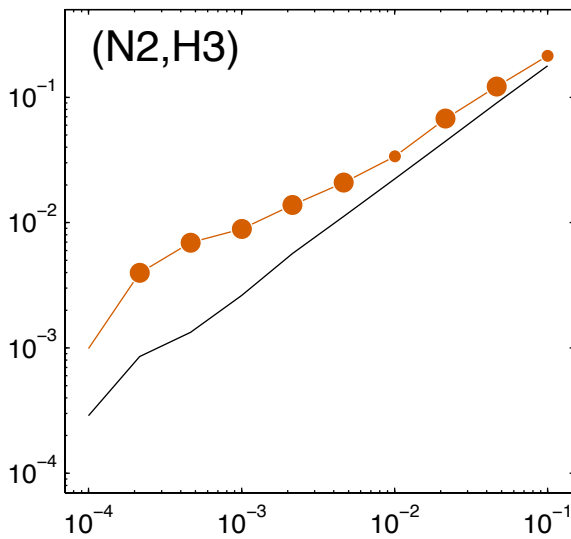

(H3,N2)

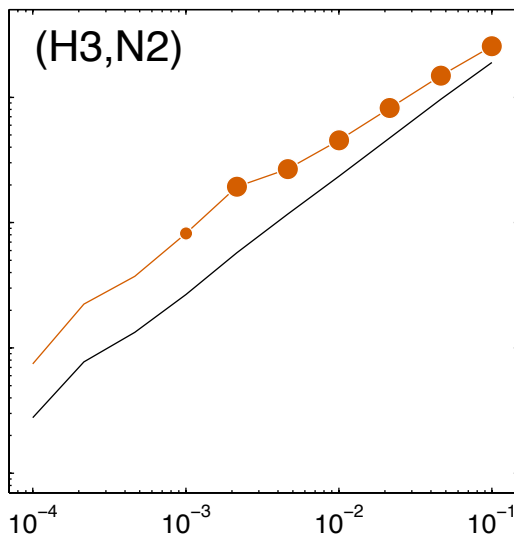

Nominal P-value

Supplement: S5 Fig — Notations as in S1 Fig. (PDF) [file pgen.1005404.s009.pdf]

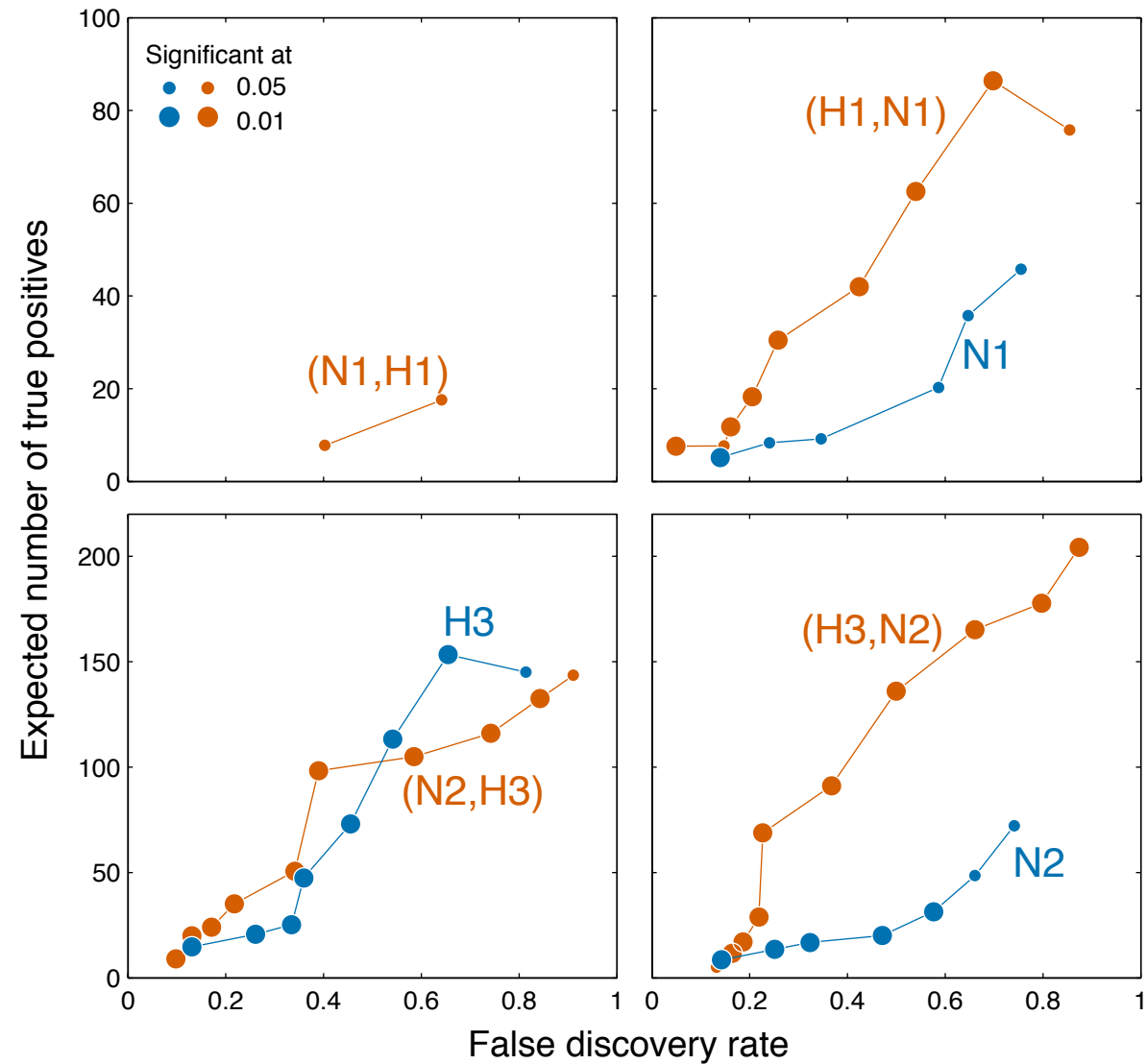

Supplement: S6 Fig — Notations as in S1 Fig. (PDF) [file pgen.1005404.s010.pdf]
